# Brain regulation training improves emotional competences in patients with alcohol use disorder

**DOI:** 10.1093/scan/nsae048

**Published:** 2024-06-25

**Authors:** Ramona L Hack, Martin Aigner, Michael Musalek, Richard Crevenna, Lilian Konicar

**Affiliations:** Department of Child and Adolescent Psychiatry, Medical University of Vienna, Vienna 1090, Austria; Anton-Proksch-Institute, Vienna, Vienna 1230, Austria; Clinical Department of Psychiatry and Psychotherapeutic Medicine, University Hospital Tulln, Tulln 3430, Austria; Anton-Proksch-Institute, Vienna, Vienna 1230, Austria; Department of Physical Medicine, Rehabilitation and Occupational Medicine, Medical University of Vienna, Vienna 1090, Austria; Department of Child and Adolescent Psychiatry, Medical University of Vienna, Vienna 1090, Austria

**Keywords:** EEG, neurofeedback, alcohol use disorder, brain regulation, addiction, emotional competences

## Abstract

Alcohol use disorder (AUD) is defined as the impaired ability to stop or control alcohol use despite adverse social, occupational, or health consequences and still represents one of the biggest challenges for society regarding health conditions, social consequences, and financial costs, including the high relapse rates after traditional alcohol rehabilitation treatment. Especially, the deficient emotional competence in AUD is said to play a key role in the development of AUD and hinders the interruption of substance compulsion, often leading to a viscous circle of relapse. Although the empirical evidence of a neurophysiological basis of AUD is solid and increases even further, clinical interventions based on neurophysiology are still rare for individuals with AUD. This randomized controlled trial investigates changes in emotional competences, alcohol-related cognitions, and drinking behavior before and after an established alcohol rehabilitation treatment (control group: *n*_CG_ = 29) compared to before and after an optimized, add-on neurofeedback (NF) training (experimental group: *n*_EG_ = 27). Improvements on the clinical–psychological level, i.e. increases in emotional competences as well as life satisfaction, were found after the experimental electroencephalography (EEG) NF training. Neurophysiological measurements via resting-state EEG indicate decreases in low beta frequency band, while alpha and theta bands remained unaffected.

## Introduction

Still in 2023, the harmful use of alcohol is one of the leading risk factors for public health and is linked to higher mortality globally (5.3% deaths worldwide) according the “Global status report on alcohol and health” from the World Health Organization (WHO 2021). In the European Union, nearly one in five adults (19%) and more than one-third (37%) of adolescents aged between 15 and 16 years reported heavy episodic drinking at least once a month in 2019 (OECD 2021). This harmful use of alcohol increased drastically during COVID-19 quarantine, especially in older individuals, essential workers, parents, and individuals with higher depression, anxiety, and impulsivity scores (Sallie et al. 2020). Taking into account the long-term impact of increased alcohol consumption during the pandemic, morbidity and mortality rates are also expected to rise (Julien et al. 2022).

Clinically, the dependence on alcohol is described with high concordance as (F10.2) Alcohol Dependence Syndrome in the *International Statistical Classification of Diseases and Related Health Problems*, 10th revision (ICD-10, WHO) (World Health Organization 1992, Grant 1993, Yoshimura et al. 2016) and in its equivalent the *Diagnostic and Statistical Manual of Disorders*, 4th revision [DSM-IV, American Psychiatric Association (APA)] (Bell 1994). While nearly all of the criteria for alcohol dependence [i.e. (i) a strong desire or sense of compulsion to use alcohol, (ii) impaired capacity to control alcohol, (iii) preoccupation with alcohol use, (iv) evidence of tolerance, (v) physiological withdrawal state when alcohol use is reduced or ceased, and (vi) persistent alcohol use despite clear evidence of harmful consequences] are similarly contained in the related subscales of the ICD-10 and DSM-IV, the definitions regarding “abuse” (DSM-IV) and “harmful use” (ICD-10) contain distinct items (Rapaport et al. 1993). However, as the distinction between alcohol abuse and alcohol dependence of the DSM-IV (Bell 1994) was already dissolved and merged in the current DSM-V (O’Brien 2011) into the term “alcohol use disorder” (AUD) and discussed also for the upcoming ICD-11(Saunders et al. 2019), as well as widely used in clinical research (Jason et al. 2012, Saunders et al. 2019, Witkiewitz et al. 2019), this term will be used henceforth.

AUD is a multifactorial disorder and is caused by a complex combination of interactions between physiological, psychological, and social factors (Rangaswamy and Porjesz 2014, Enoch and Albaugh 2017, Rawls et al. 2021). The clinical symptoms of AUD are condensed in a triadic symptom cluster, linking deficits on the cognitive-behavioral level (craving and impulsive drinking behavior) (Lejuez et al. 2010) to shortcomings in the affective domains (emotional competences) (Jakubczyk et al. 2018) to altered neurophysiological processes (structural and functional changes of the brain) (Schepis et al. 2011, Bates et al. 2013, Rawls et al. 2021).

On the cognitive-behavioral level, AUD is characterized by a strong desire or sense of compulsion to consume alcohol (craving; thoughts about drinking), while the ability to control alcohol consumption is impaired (impulsive drinking behavior) (Yoshimura et al. 2016, Jakubczyk et al. 2018). Similarly to findings in the forensic population (Konicar et al. 2015, Fielenbach et al. 2017, 2018), patients with AUD tend to show risky behavior and cannot stop or control impulses of drinking (Czapla et al. 2016), while emotional perception abilities seem to be impaired.

Regarding the affective shortcomings, deficits in emotional competences (EC; i.e. emotional intelligence) involve a variety of domains, such as emotion perception and awareness, emotion recognition, bodily experience, emotional imaginary, emotional overflow, lack of emotions, emotional self-efficacy, acceptance of emotions, and emotional self-control/emotion regulation (Saarni 1999, Mayer et al. 2000, Mathews et al. 2016). These domains could be influenced by substance intake, as various legal and illegal substances immediately increase the experience of pleasant emotional states (e.g. euphoria, happiness, feeling of interpersonal closeness and attachment), while alleviating unpleasant emotional states (e.g. anxiety, sadness, and depression) or relieving the absence/lack of emotional experience (i.e. often reported feeling as being “cut off from feelings and relationships”) (Khantzian 1990). Considering this mechanism, it is obvious that the positive emotional expectancies related to the drug intake reinforce and accelerate the next intake (Kober and Bolling 2014). Whether the relationship between emotional competences and substance abuse could be explained by either reward- or rather relief-drinking mechanisms is still an open discussion (Huang et al. 2018). A common key factor is represented by the process of emotion regulation (closely linked to the regulation of drinking behavior), as deficits in emotion regulation in childhood and adolescence are revealed as a risk factor in the development of a substance abuse disorder (SUD) (Kober and Bolling 2014, Huang et al. 2018). In summary, deficits in emotional competences (especially deficits in the regulation of emotions) were repeatedly shown to be linked to substance abuse (e.g. tobacco, marijuana, and alcohol) (Riley and Schutte 2003, Dvorak et al. 2014, Mikolajczak et al. 2015, Mohagheghi et al. 2015) and were related to higher impulsivity, resulting in higher relapse rates (Petit et al. 2015, Jakubczyk et al. 2018, Ottonello et al. 2019) and even predict alcohol relapse (Fox et al. 2008, Berking et al. 2011, Garland 2021).

On a neurophysiological level, magnetic resonance imaging studies indicate atrophy in various brain regions involved in cognitive, behavioral, and emotional processing and regulation in patients with AUD (Cui et al. 2015, Gilmore and Fein 2012, Wang et al. 2018), especially in frontal and parietal areas (Cardenas et al. 2007). Moreover, early-onset drinking in adolescents is associated with increased resting-state functional connectivity between the nucleus accumbens and prefrontal parietal, and medial temporal regions (Weissman et al. 2015). On a genetic level, potential genetic influences were revealed, as pre-existing diagnosis of AUD of family members was found to be associated with decreased functional connectivity between frontal, cerebellar, and parietal regions in offspring (Herting et al. 2011, Wetherill et al. 2012).

In a similar vein, electroencephalography (EEG) studies report alterations in the resting-state EEG (rsEEG), revealing increases in the alpha frequency band (Stenberg 1992), as well as in slow alpha frequency (7.5–9 Hz) in participants with AUD and/or with a family history of alcoholism (Ehlers and Phillips 2003, Porjesz et al. 2005). Increased activity in beta frequency bands, especially in slow beta frequency (beta 1: 12–16 Hz), were found in male, high-risk subjects, offspring of male alcoholics, and male alcoholics (Rangaswamy et al. 2002; Rangaswamy et al. 2004). The increased slow beta power in resting EEG in AUD may reflect a hyperarousal of the central nervous system (Campanella et al. 2009), indicating a risk marker for developing alcoholism as predictive endophenotype.

Taken together, besides inconsistent findings in rsEEG regarding alpha, theta, and delta frequency bands (Liu et al. 2022), the most common finding, reflecting a broad agreement in literature, is a marked increase of the EEG beta band at all regions over the scalp in individuals with AUD at rest, but most important at parietal and central regions (Kamarajan 2019, Liu et al. 2022, Mumtaz et al. 2018, Porjesz et al. 2005; Rangaswamy and Porjesz 2014).

Although the empirical evidence of a psychophysiological basis of AUD is solid and increases even further (O’daly et al. 2012, Fitzpatrick and Crowe 2013, Wilcox et al. 2016), clinical interventions based on psychophysiology are still rare in individuals with AUD [see reviews on design, methodology, and application in Niv (2013) and Marzbani et al. (2016)]. One psychophysiological approach, which has been proven as a suitable clinical treatment for alcohol dependence (i.e. AUD) is brain regulation training via EEG-based neurofeedback (NF). Here, especially the training of alpha and theta EEG frequency bands named as the “Peniston Protocol” (Peniston and Kulkosky 1989, 1991, 1999) or the further developed “Scott–Kaiser Protocol” (Scott et al. 2005), demonstrated improvements in relaxation, craving, depression, post-traumatic stress disorder symptoms, and personality traits. Furthermore, decreased relapse rates were found after these EEG NF trainings (Saxby and Peniston 1995, White 1999, Callaway and Bodenhamer-Davis 2008, Lackner et al. 2016, Dalkner et al. 2017). The great success of this special alpha/theta (AT) NF protocol is seen in the generation of a state of deep relaxation, a state of reverie (Peniston and Kulkosky 1991). This normally not conscious mental state, before falling asleep, is associated with a decrease in alpha band and an increase in theta band, with the intersection (when the theta band becomes more dominant than the alpha band) named as “alpha/theta crossover.” Using EEG NF, this state could be entered consciously by increasing the theta to alpha ratio, which is said to facilitate not only internal visual imagery but also the emotional imagery, linked to improved processing of emotional conflicts and related adaptations in behaviors and attitudes (Egner and Gruzelier 2004, Egner et al. 2002; J. Gruzelier 2009, White 1999). Increases in the alpha and theta bands parallel to decreases in depression scores were found after 30 NF sessions (Peniston and Kulkosky 1991, 1999). In a later study, Peniston reported that during AT sessions in which visual imagery was reported, significant amplitude increases occurred in both the theta and beta ranges but not in the EEG alpha band. Follow-up measures after 13 months demonstrated significant lower relapse rates in the experimental NF group compared to the control group (CG) (Peniston and Kulkosky 1999). In recent studies, after 12 NF sessions, an increase in the alpha and theta bands occurred, and this increase was associated with decreased avoidant and stress-related personality traits (Dalkner et al. 2017) and clinical changes (Lackner et al. 2016) in patients with AUD.

Besides alpha and theta frequency bands as efficient brain training targets (Egner et al. 2002; Gruzelier et al. 2006), the sensorimotor rhythm (SMR) has been found to be significantly effective in reducing anxiety and related cortisol levels (Gadea et al. 2020) and improving emotional processes (Gruzelier 2014a), memory, and attentional performance (Kober et al. 2015), as well as spatial abilities and creativity (Doppelmayr and Weber 2011). Moreover, Gadea et al. (2020) reported increases in the SMR band after SMR NF training, parallel to decreases in anxiety after only one session of SMR up and theta down NF training (Gadea et al. 2020). Regarding the balancing effect on executive functions and behavior, a recent meta-analysis highlighted SMR NF training as one of the three standard NF training protocols for Attention Deficit Hyperactivity Disorder (ADHD) (Enriquez-Geppert et al. 2019) with large effect sizes for inattention, impulsivity, and sleep onset latency (Arns et al. 2009, 2012, 2014, Van Doren et al. 2019), while some other studies could not attribute the findings of decreased impulsivity and craving directly to the SMR NF (Fielenbach et al. 2018, 2019). Based on several clinical findings of comorbid ADHD in patients with substance use disorder, the Peniston Protocol was further modified by Scott–Kaiser, who combined the EEG beta band and the SMR with the established AT training (Scott and Kaiser 1998; Scott et al. 2005). This protocol consisted of 10–20 sessions of beta/SMR training with eyes open, followed by 30 sessions of AT training. After this modified Scott–Kaiser NF training, improvements in attention, general distress, and increased abstinence rates were reported in patients with SUD (Scott and Kaiser 1998). This finding was supported by a follow-up study with patients with mixed SUD (including alcohol, heroin, cocaine, and methamphetamine). Compared to the CG, receiving only conventional treatment, the NF group (Scott–Kaiser Protocol as an add-on to the conventional treatment) was longer abstinent within a year after treatment, stayed longer in treatment in general, and yielded lower dropout rates (Scott et al. 2005). The efficacy of the Scott–Kaiser modification of the Peniston Protocol was confirmed by a multiplicity of studies with patients with SUD and demonstrated solid improvements in depression, anxiety, somatic symptoms, craving, and general mental health, across varying conditions (varying electrode position, number of sessions, etc.) (Burkett et al. 2005, Dehghani-Arani et al. 2013, Rostami and Dehghani-Arani 2015). For this reason, Peniston’s AT protocol and the Scott–Kaiser modification can both be classified as Level 3 “probably efficacious” under the terms of combining the NF training with residential therapy (Sokhadze et al. 2008).

Based on these previous achievements and recommendations (Sokhadze et al. 2008, Dousset et al. 2020, Russo et al. 2023), we assume that an EEG NF training approach combining the most successful brain wave targets, i.e. AT and SMR, yields synergetic effects for application in patients with AUD.

Targeting the different levels of the triadic clinical symptom cluster, we primarily expect clinical–psychological improvements in patients with AUD on the “emotional/affective level” (i.e. increases in emotion regulation, known as the most important emotional deficits in AUD, as well as increases in emotional imaginary, parallel to improvement regarding the potential lack of emotions) after the optimized SMR—AT NF training [as an add-on to treatment as usual (TAU); TAU + NF] compared to a control TAU group (TAU only).

Secondarily, we expect further clinical–psychological improvements on the cognitive-behavioral level i.e. reductions in alcohol-related cognitions and impulsive drinking behavior in the experimental TAU + NF group, compared to the TAU only CG after the interventions.

On a neurophysiological level, we primarily expect changes in centroparietal low beta frequency (i.e. a possible balancing effect on the reported increased low beta in individuals with AUD) after the NF training in the experimental group (EG) compared to after TAU in the CG.

In an exploratory manner, we also target changes in further clinical symptoms of AUD (depression, quality of life, cognitive strategies, stress coping, and impulsivity), as well as possible further changes on the neurophysiological level (i.e. changes in alpha and theta frequencies from before to after the interventions in the EG and the CG).

## Materials and methods

### Subjects and study design

**Figure 1.**
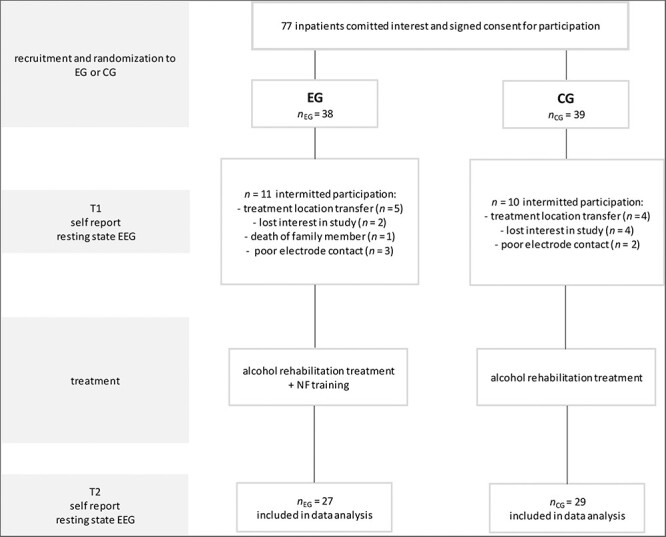
Study design and procedure.

In total, 77 participants with the main diagnosis F10.2 Alcohol Dependence Syndrome (World Health Organization 1992) were recruited and randomly assigned (by tossing a coin) to either a CG (TAU), receiving the established alcohol rehabilitation program (ARP), or to an EG, receiving an adopted and optimized EEG-based NF training in addition to the established ARP (see Fig. 1). Individuals with epilepsy, organic brain damage, and psychosis were excluded from the study. Study inclusion and intervention began after physical alcohol detoxification, while subjects in both groups continued pharmacological treatment. Before (T1) and after (T2) the therapeutic interventions, brain activity (via rsEEG) and clinical symptomatology (via self-reports) were measured in both groups (EG and CG) (see Fig. 1). Eight individuals dropped out after randomization at T1 in each group due to different reasons (e.g. location transfer, lost interest in study, or death of a family member).

Given the study design with two EGs (EG and CG) and two measurement time points (pre = T1; post = T2) and an assumed correlation of *r* = 0.50, a repeated-measures analysis of variance (rmANOVA) resulted in a required minimum sample size of *N* = 54 to detect a medium-sized treatment effect of *f* = 0.25, using G*Power version 3.1.9.4 (Faul et al. 2007).

All pre/post measures, as well as all clinical interventions, took place at the psychological laboratory of the Anton-Proksch-Institute in Vienna, Austria. This study was conducted according to the guidelines of the Declaration of Helsinki and approved by the Ethics committee of the University of Vienna (EK Nr 1203/2015).

### Materials

#### Clinical–psychological symptomatology

Clinical–psychological symptoms of AUD were collected using the following self-reports:

To examine changes on the affective/emotional level, i.e. emotional competence, the “Scales for Experiencing Emotion” (SEE) (Behr and Becker 2004) were used. This self-report measures how individuals perceive, evaluate, and deal with their own feelings on seven subscales, namely: (i) “Acceptance of One’s Own Emotions” (SEE_AE), (ii) “Overflow of Emotions” (SEE_OE), (iii) “Lack of Emotions” (SEE_LE), (iv) “Bodily Experience of Emotions” (SEE_BE), (v) “Self-Control” (SEE_SC), (vi) “Emotional Imaginary” (SEE_EI), and (vii) “Emotion Regulation” (SEE_ER).

To quantify changes on the cognitive-behavioral level, we used the “Obsessive-Compulsive Drinking Scale” (OCDS) (Anton et al. 1996), with the subscales (i) “Alcohol-related Cognitions” and (ii) “Impulsive Drinking Behavior” as our secondary outcome measure.

For further exploratory analysis, we used the “Beck Depression Inventory II Revision” (BDI II) (Beck et al. 1996) to measure the severity of depression and the “Questionnaire of Dysfunctional and Functional Self-Consciousness” (DFS) (Hoyer 2000) to shed light on (i) “Functional Cognitive Strategies” and the opposite (ii) “Dysfunctional Cognitive Strategies” before and after the therapeutic interventions. To investigate potential changes regarding stress coping strategies based on the following five subscales: (i) “Positive Strategies Total,” (ii) “Positive Strategies of Reframing,” (iii) “Positive Strategies of Distraction,” (iv) “Positive Strategies of Control,” and (v) “Negative Strategies Total,” we used the “Stress Coping Questionnaire” (SVF) (Erdmann and Janke 2008) before and after the therapeutic interventions. Possible changes in impulsivity were detected with the “Impulsive Behavior Scale” (UPPS) (Schmidt et al. 2008), including the facets (i) “Urgency,” (ii) “Lack of Premeditation,” (iii) “Lack of Perseverance,” and (iv) “Sensation Seeking.” Finally, the “Life Satisfaction Self-Report Questionnaire” (FLZ) (Fahrenberg et al. 2000) was used to index the patients’ life satisfaction on two dimensions, namely regarding the (i) “Person” (satisfaction with oneself) and the (ii) “Persons’ Health” (satisfaction with your own health).

#### rsEEG recording and data processing

rsEEG was recorded with a NeXus-32 channel Neuro-/Biofeedback System combined with BioTrace + Software (Mind Media; Herten, Netherlands). The NeXus-32 system includes 21 channels with sintered and reusable electrodes, built into the cap according to the 10–20 system (Jasper 1958, Milnik et al. 2020). Besides 19 active electrodes, two auricular reference electrodes and a ground electrode at FPz were used for all EEG measures. EEG sampling rate was 256 Hz; electrode impedances were constantly kept below 5 kΩ. For rsEEG, participants were asked to relax during two different recording settings: (i) with 10 min eyes open (EO) and (ii) subsequent 10 min eyes closed (EC). During the EO condition, a fixation cross was positioned in the middle of the screen. During the EC condition, an unpleasant sound (i.e. bird chirping) was presented in case of the occurrence of delta frequency band (1–4 Hz) to prevent patients from falling asleep.

EEG data were preprocessed and analyzed using Brain Vision Analyzer 2.2 (Brain Vision Analyzer 2.2 2006). After a notch (50 Hz) and bandpass filter (1–70 Hz), raw data were manually and semi-automatically inspected for artifacts and corrected. Subsequently, Independent Component Analysis was conducted for ocular correction, after which Fourier Transformation (10% length; Hanning Window) was applied to segmented conditions. Finally, segmented data were averaged into power spectra for each condition (T1 and T2; EO and EC) and each participant. The frequency bands of interest were extracted as recorded by the NF system as low beta (12–15 Hz), theta (4–8 Hz), and alpha (8–12 Hz) frequencies, from which mean total scores for centroparietal regions (Mean_centroparietal_ = Cz, C3, C4, Pz, P3, P4) were calculated.

### Therapeutic methods: NF training and ARP

Based on the Peniston (Peniston and Kulkosky 1999) and Scott–Kaiser protocols (Scott et al. 2005), we developed an optimized and combined EEG NF protocol. Each of the 15 NF training sessions consisted of two parts: firstly, an SMR training with EO, followed by a short break, after which an AT training with EC followed.

**Figure 2.**
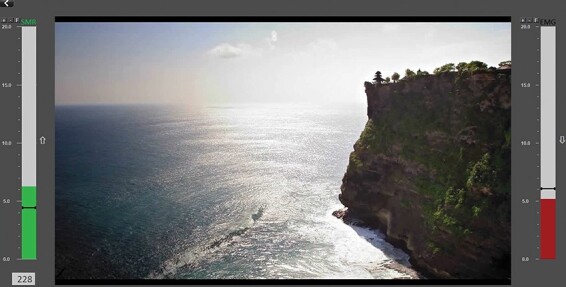
BioTrace NF screen (Mind-Media NeXus-10 MKII device; Herten, Netherlands) with visual feedback. With courtesy of Mind Media Nexus (mindmedia.com).

For SMR training (12–15 Hz), brain activity was fed back from EEG position Cz to the participants’ monitor for 20 min (as a preferred animation, e.g. waves as illustrated in Fig. 2). Every session started with a 2-min baseline recording to determine the individual threshold. In the following active regulation phases, SMR activity was displayed as a bar graph, and the participants were instructed to develop their own regulation strategy for modulating their SMR activity in such a way that the bar graph turns from red to green (i.e. SMR band power reaches the individually predefined threshold of 60% above baseline for 125 ms). Besides this, no other instruction was provided regarding the efficiency or the success of specific strategies for SMR regulation. Hence, the animation stopped when brain activity was under the predefined threshold and moved on when brain activity was over the predefined threshold. Subjects could change animation content within each session to maximize motivation and performance. An additional numerical “scoring counter” counted and displayed the current NF regulation success, i.e. it increased whenever SMR increased and remained above threshold (1 point = 500 ms above threshold; see in the left corner in Fig. 2). The general verbal NF instruction emphasized that muscular strategies (i.e. muscular tension–relaxation measured via a tension bar graph, based on a predefined movement artifact threshold) or respiratory strategies disturb NF performance. As demonstrated in Figure 2, moving waves indicated successful regulation of SMR frequency, while a still animation (”frozen” waves) indicated a non-successful regulation of SMR frequency. Artifacts (muscle/movement thresholds) were visualized by an electromyography (EMG) bar at the right side of the screen and the bar at the left side of the screen indicated the SMR amplitude.

After a short break, the second part of the optimized, combined EEG NF started, namely 20 min of AT training. Here, the brain activity of the alpha band (8–12 Hz), respectively, of the theta band (4–8 Hz), was fed back from the EEG position Pz. During the AT training, the participants were instructed to close their eyes, imagine a pleasant situation, and listen to a relaxing sound (e.g. pleasant melody). Similarly, here, a 2-min baseline was recorded at the beginning of the session to define the individual threshold. Afterward, feedback was given acoustically as music, which varied in volume depending on whether the alpha band (60%–70% above baseline for 125 ms) or the theta band (40%–60% above baseline for 125 ms) reached the predefined thresholds. Standard online processing settings of AT training included a beta inhibit (13–36 Hz) coupled with another unpleasant sound as acoustic feedback in case of exceeding 5 µV, as well as a delta/theta (2–6 Hz) inhibit coupled in the same vein with acoustic feedback, namely a bird chirping, whenever the predefined threshold of 20 µV was reached, following the recommended default online processing steps (Mind Media; Herten, the Netherlands).

Both SMR and AT trainings were performed with the Mind-Media NeXus-10 MKII device (Mind Media; Herten, the Netherlands) with a sampling rate of 256 Hz. The reference electrode was placed on the left earlobe, while the ground electrode was placed on the right earlobe. During each training session, artifacts through muscle tension or eye movement were inhibited at a maximum threshold of 1.5 µV, and feedback was given visually (during SMR training) or acoustically (during AT training) (see Fig. 2).

For comparison, an established ARP served as “TAU” for the CG. The established ARP is a multimodal, designed, structured intervention with the following therapeutic units: psychotherapy, art therapy, or medical/physical training (Musalek 2011). Self-reports and rsEEG were compared before and after the optimized EEG NF training in addition to the ARP of the EG (EG: NF + ARP) to self-reports and rsEEG before and after the ARP of the CG (CG: ARP only).

## Statistical analysis

All statistical analyses were conducted using SPSS 27 (IBM SPSS Statistics 27 2011). As rmANOVA is seen as generally robust and the Type I error and power of the F-statistics are not altered by the violation of normality when the sphericity assumption is met (Blanca et al. 2023), we maintained a parametric statistical procedure even in cases of altered normality to provide robust results.

Consequently, for the primary clinical–psychological outcome, namely emotional competence, an rmANOVA with within-subject factor Time (pre = T1/ post = T2 measures of the SEE subscales “Emotion Regulation,” Emotional Imaginary, and Lack of Emotions) and between-subject factor Group (EG/CG) was applied. For corrections regarding multiple testing of the three SEE subscales, a Bonferroni-adjusted significance level of *α* = .0167 (.05/3 = .0167 as the Bonferroni-adjusted alpha level) was considered. In addition, a total score of emotional competence was computed out of all SEE subscales (Total Emotional Competence = SEE_AE − SEE_OE − SEE_LE + SEE_BE + SEE_SC + SEE_EI + SEE_ER) as a general indicator for comparison reasons and provided in the Supplementary material (C) “Detailed Analysis of ‘Emotional Competence’ Total Score.”

Regarding the secondary clinical–psychological outcome, namely alcohol-related cognitions and impulsive drinking behavior, an rmANOVA with within-subject factor Time (pre = T1/ post = T2 measures of the OCDS subscales “Alcohol-related Cognitions” and “Impulsive Drinking Behavior”) and between-subject factor Group (EG/CG) was applied. For corrections regarding multiple testing of the two OCDS subscales, a Bonferroni-adjusted significance level of *α* = .025 (.05/2= .025 as the Bonferroni-adjusted alpha level) was considered.

For exploratory analysis regarding other clinical–psychological symptoms of AUD, similarly an rmANOVA with within-subject factor Time (pre = T1/ post = T2 measures of in total 14 subscales of the psychological questionnaires BDI, DFS, UPPS, FLZ, and SVF) and between-subject factor Group (EG/CG) were applied. In addition to reporting uncorrected *P* values regarding the listed exploratory analysis, the Benjamini–Hochberg (BH) (Benjamini and Hochberg 1995) corrected *P* values are provided.

For analysis on the neurophysiological level, in a similar vein, an rmANOVA was applied with the within-subject factor Time (pre = T1/ post = T2 measures of centroparietal low beta band with EO and EC) and between-subject factor Group (EG/CG). For corrections regarding multiple testing of the two recording settings (EO and EC), a Bonferroni-adjusted significance level of *α* = .025 (.05/2 = .025 as the Bonferroni-adjusted alpha level) was considered.

Regarding further exploratory analysis on the neurophysiological level, an rmANOVA was applied with the within-subject factor Time (pre = T1/ post = T2 measures of centroparietal alpha band with EO and EC) and between-subject factor group (EG/CG). In the same vein, an rmANOVA was used to detect exploratory changes in rsEEG theta band with the within-subject factor Time (pre = T1/ post = T2 measures of centroparietal theta band with EO and EC) and between-subject factor Group (EG/CG). Corrections for multiple testing regarding the exploratory analysis on the neurophysiological level yielded similar results and were therefore omitted in the following. Centroparietal regions included the electrode positions: Cz, C3, C4, Pz, P3, and P4.

Pearson’s correlations were used to exploratory investigate the relationships between changes in clinical–psychological symptomatology [post (T2) minus pre (T1)] and changes in rsEEG [post (T2) minus pre (T1)]. For all exploratory analysis, *P* values ≤ .05 are considered as statistically significant. In addition, effect sizes as measured by Cohen’s *d* (Cohen 2013) are provided.

For comparison reasons regarding the reporting of NF studies, the “CRED-nf best practices checklist” [Consensus on the Reporting and Experimental Design of clinical and cognitive-behavioral Neurofeedback studies (Ros et al. 2020)] is provided in Supplementary material (A). An overview over the course of NF learning as well as NF regulation strategies is provided in Supplementary material (D) in a first descriptive, basic manner.

## Results

### Clinical sample

The final sample consisted of 56 inpatients (33 male, 23 female) with a mean age of *M*_Age_ = 40.78 years (SD_Age_ = 10.79) in the EG (*n*_EG_ = 27) and a mean age of *M*_Age_ = 48.79 (SD_Age_ = 11.16) in the CG (*n*_CG_ = 29), with comparable grades of severity of alcohol dependence and similar amounts of psychological and physical comorbidities [for more information, see Supplementary material (B) “Detailed Description of Clinical Sample”].

### Clinical–psychological symptomatology

#### Emotional competence

**Figure 3.**
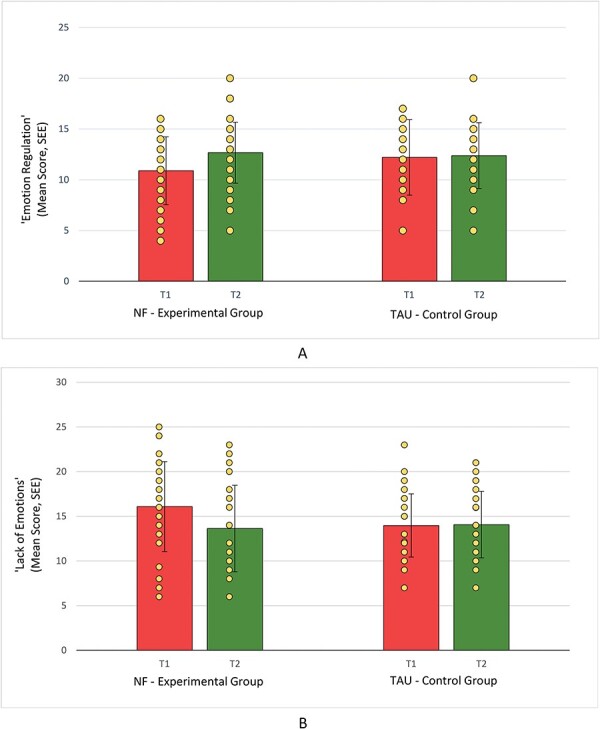
Increases in subscales “Emotion Regulation” (A) and decreases in the “Lack of Emotion” (B) from before intervention (T1) to after intervention (T2) in the NF (*n*_NF_ = 27) EG compared to the TAU (*n*_TAU_ = 29) CG in subscales of SEE [Scales for Experiencing Emotion (Behr and Becker 2004)].

rmANOVA revealed a significant main effect of Time with lower scores of the SEE subscale “Lack of Emotions” at T2 compared to T1 [*F*(1, 54) = 5.64; *P* = .021; *η*^2^= 0.095] and higher scores of the subscale “Emotion Regulation” at T2 compared to T1 [*F*(1, 54) = 9.48; *P* = .003; *η*^2^= 0.149]. Due to significant interactions Time*Group [“Lack of Emotions”: *F*(1,54) = 6.60; *P* = .013; *η*^2^= 0.109; “Emotion Regulation”: *F*(1,54) = 6.43; *P* = .014; *η*^2^= 0.106], pairwise post-hoc tests were conducted and revealed a significant increase in “Emotion Regulation” (*t*_(26)_ = −3.63, *P* = .001) from before (*M* = 10.89, SD = 3.34) to after intervention (*M* = 12.67, SD = 3.72), as well as a significant decrease in “Lack of Emotions” (*t*_(26)_ = 3.12, *P* = .004) from before (*M* = 16.09, SD = 5.03) to after intervention (*M* = 13.63, SD = 4.84) in the EG but no changes from before to after intervention in the CG, as depicted in Fig. 3A and B. Bonferroni corrections were applied and confirmed the significant interactions for “Lack of Emotions” (*P* = .013 < .0167_adjusted alpha level_) and “Emotion Regulation” (*P* = .014 < .0167_adjusted alpha level_). The effect sizes, as measured by Cohen’s *d*, were *d* = 0.60 for the decrease in “Lack of Emotions” and *d* = −0.69 for the increase in “Emotion Regulation,” indicating medium effects. No significant differences between the EG and the CG were found at baseline (T1).

#### Alcohol-related cognition and impulsive drinking behavior

**Figure 4.**
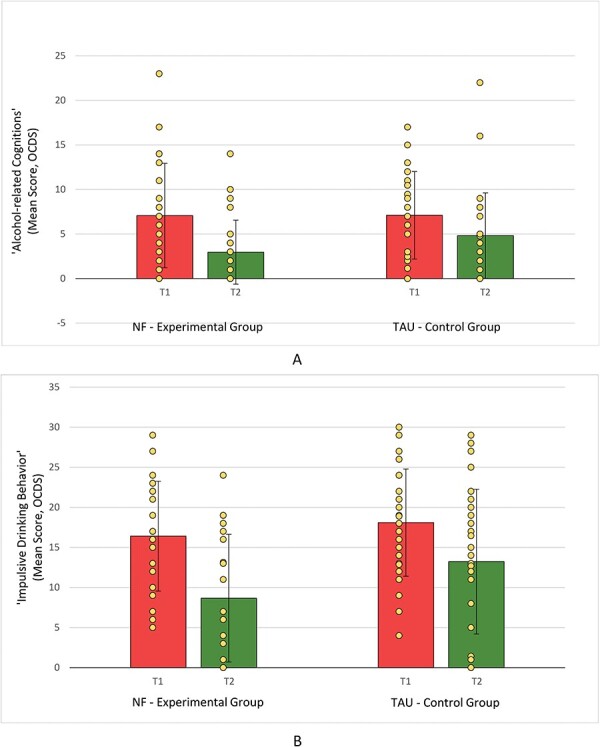
Main effect of time: decreases in subscales “Alcohol-related Cognitions” (A) and “Impulsive Drinking Behavior” (B) [Obsessive-Compulsive Drinking Scale (OCDS) (Anton et al. 1996)] from before intervention (T1) to after intervention (T2) of NF (*n*_NF_ = 27) EG and TAU (*n*_TAU_ = 29) CG.

As depicted in Fig. 4, besides a significant main effect of Time with decreased scores in the OCDS subscale “Alcohol-related Cognitions” [*F*(1,54) = 27.77; *P* < .001; *η*^2^ = 0.340] at T2 (*M* = 3.92, SD = 4.32) compared to T1 (*M* = 7.09, SD = 5.34), as well as a decrease in the subscale “Impulse Drinking Behavior” [*F*(1,54) = 35.09; *P* < .001; *η*^2^ = 0.394] at T2 (*M* = 11.03, SD = 8.76) compared to T1 (*M* = 17.23, SD = 6.76), no significant interactions were found (before as well as after Bonferroni corrections were applied). No significant differences between the groups were found at baseline—before treatment [“Alcohol-related Cognition”: *t*_(54)_ = 0.20, *P* = .98; “Impulsive Drinking Behavior”: *t*_(54)_ = 0.93, *P* = .36], while after treatment, a group difference trend could be observed for the subscale “Impulsive Drinking Behavior” [*t*_(54)_ = 2.00, *P* = .051] but not for “Alcohol-related Cognitions” [*t*_(54)_ = 1.63, *P* = .109].

#### Exploratory analysis of additional clinical and psychological measures

Exploratory analysis regarding further clinical and psychological symptoms points toward significant improvements in “Life Satisfaction” related to health and personal aspects in the FLZ (Fahrenberg et al. 2000) from before to after intervention in the EG, compared to the CG (considering BH-adjusted *P*-values, as provided in Table 1). Further improvements were found in the subscales “Positive Strategies of Control” [SVF (Erdmann and Janke 2008)], “Lack of Premediation” [UPPS (Schmidt et al. 2008)], “Lack of Perseverance” [UPPS (Schmidt et al. 2008)], “Positive Strategies Total” [SVF (Erdmann and Janke 2008)], and “Negative Strategies Total” [SVF (Erdmann and Janke 2008)] (considering uncorrected *P*-values, as summarized in Table 1).

**Table 1.** Exploratory analysis of additional clinical–psychological measures.

	EG (*n*_EG_ = 27)		CG (*n*_EG_ = 29)					
Clin.-Psy. questionnaires / subscales	T1 *M*(SD)	T2 *M*(SD)	T1 *M*(SD)	T2 *M*(SD)	*F* (1, 54)	*P* values Uncorrected	*η^2^*	*P* values BH-adjusted
FLZ: Health	29.85 (8.87)	35.83 (7.60)	32.38 (8.01)	33.68 (8.48)	1, 05	.000***	0.16	.03*
FLZ: Person	32.00 (8.13)	35.48 (9.77)	35.83 (7.64)	34.96 (9.04)	9, 14	.000***	0.11	.03*
SVF: Positive Strategies of Control	14.34 (3.83)	15.20 (2.95)	15.36 (3.98)	14.54 (3.16)	5, 36	.02*	0.09	.06
UPPS: Lack of Premediation	22.00 (5.16)	21.44 (4.76)	24.03 (4.41)	24.40 (4.97)	0, 96	.02*	0.09	.06
UPPS: Lack of Perseverance	19.33 (4.66)	18.33 (4.16)	19.80 (4.81)	20.66 (5.02)	5, 33	.03*	0.09	.06
SVF: Positive Strategies Total	12.17 (2.58)	13.15 (2.49)	12.65 (2.85)	12.42 (2.87)	4, 77	.03*	0.08	.06
SVF: Negative Strategies Total	12.30 (5.32)	10.36 (5.19)	10.79 (4.21)	10.46 (4.29)	5, 07	.03*	0.12	.06
BDI	16.37 (10.92)	7.87 (7.93)	17.60 (10.93)	13.38 (11.66)	4, 19	.05	0.07	.09
SVF: Positive Strategies of Reframing	9.93 (3.94)	11.13 (4.34)	11.26 (3.56)	11.26 (4.48)	1, 96	.17	0.04	.24
DFS: Dysfunctional Strategies	45.02 (11.37)	39.29 (11.44)	44.94 (8.27)	42.14 (9.09)	1, 95	.17	0.04	.24
DFS: Functional Strategies	26.59 (5.06)	28.48 (4.93)	27.07 (5.85)	27.46 (5.30)	1, 77	.19	0.03	.24
UPPS: Urgency	31.11 (7.86)	28.96 (7.27)	29.70 (6.03)	28.74 (5.97)	0, 84	.36	0.02	.42
SVF: Positive Strategies of Distraction	11.16 (3.17)	12.09 (3.41)	9.97 (3.84)	10.43 (3.92)	0,32	.57	0.006	.61
UPPS: Sensation Seeking	30.30 (9.32)	31.30 (8.15)	27.79 (7.20)	27.69 (7.96)	1, 23	.68	0.01	.68

EG—NF training, CG—TAU, T1: before intervention, T2: after intervention. BDI-II, Beck Depression Inventory II Revision (Beck et al. 1996); DFS, Questionnaire of Dysfunctional and Functional Self-Consciousness (Hoyer 2000); SVF, Stress Coping Questionnaire (Erdmann and Janke 2008); UPPS, Impulsive Behavior Scale (Schmidt et al. 2008); FLZ, Life Satisfaction Self-Report Questionnaire (Fahrenberg et al. 2000). *  *P* < .05, **  *P* < .01, ***  *P* < .001.

### Neurophysiological measures

#### Low beta rsEEG analysis

rmANOVA regarding the third of the triadic symptom cluster levels of AUD, namely, the neurophysiological level, showed a significant main effect of Time [EO: *F*(1, 54) = 6.06; *P* = .017; *η*^2^ = 0.101; EC: *F*(1, 54) = 8.96; *P* = .004; *η*^2^ = 0.142], indicating decreases in low beta band in centroparietal regions from T1 (EO: *M* = 0.62, SD = 0.57; EC: *M* = 0.99, SD = 0.81) to T2 (EO: *M* = 0.45, SD = 0.45; EC: *M* = 0.66, SD = 0.66). Due to significant Time*Group interactions [EO: *F*(1, 54) = 5.76; *P* = .020; *η*^2^ = 0.096; EC: *F*(1, 54) = 51.42; *P* = .049; *η*^2^ = 0.070], post-hoc tests were conducted and revealed a significant decrease in the experimental NF group [EO: *t*_(26)_ = 2.91, *P* = .007; EC: *t*_(26)_ = 2.93, *P* = .007] from before (EO: *M* = 0.76, SD = 0.64; EC: *M* = 1.27, SD = 0.85) to after intervention (EO: *M* = 0.42, SD = 0.30; EC: *M* = 0.71, SD = 0.67), while no significant changes from before to after intervention were found in the CG (neither in the EO nor in the EC condition). No significant baseline (T1) differences between the EG and the CG were found in the EO condition, but differences were found in the EC condition (EC: *t*_(54)_ = −2.65, *P* = 0.011). Bonferroni corrections for the two recording settings (EO and EC) were applied for multiple testing, resulting in an adjusted alpha level of (.05/2) *P* = .025, confirming the significant Time*Group interaction in the EO condition [EO: *F*(1, 54) = 5.76; *P* = .020 < 0.025_adjustedBonferroni_]. The related effect size, as measured by Cohen’s *d*, was *d* = 0.56 for the decreases in centroparietal low beta band from before to after the NF intervention in the EG, as a medium effect.

#### Exploratory rsEEG analysis

Regarding changes in alpha band from before to after the interventions, repeated-measures analysis yielded a trend for the main effect of Time for the EC condition [EC: *F*(1, 54) = 3.93; *P* = .051; *η*^2^ = 0.068], while the EO condition [EO: *F*(1, 54) = 1.97; *P* = .166; *η*^2^ = 0.035] remained unchanged.

Due to a trend regarding the interaction Time*Group in the EO condition [EO: *F*(1, 54) = 3.37; *P* = .072; *η*^2^ = 0.059; EC: *F*(1, 54) = 0.282; *P* = .60; *η*^2^ = 0.005], post-hoc tests were conducted and confirmed the trend, i.e. a slight decrease in alpha band in the experimental NF group [EO: *t*_(26)_ = 1.81, *P* = .082] from before (*M* = 1.29, SD = 1.31) to after intervention (*M* = 0.81, SD = 0.71), while neither significant changes nor trend from before to after intervention could be observed for the EC condition and for the CG (neither in the EO nor in the EC condition). Significant baseline (T1) differences between the EG and the CG were found in the EC condition [EC: *t*_(54)_ = −2.24, *P* = .029] and a trend for the EO condition [EO: *t*_(54)_ = −1.82, *P* = .074].

Exploratory analysis regarding changes of rsEEG theta frequency band showed no significant main effects of Time, nor significant interactions or trends from before to after interventions between the experimental NF and the CG.

#### Exploratory analyses: relationships between rsEEG measures and clinical–psychological symptomatology

No significant relationships between the reported changes of the primary clinical**–**psychological indicators (increases in “Emotion Regulation” and decreases in “Lack of Emotion”) and the changes on the primary neurophysiological level (decreases in low beta, EO) from before to after interventions were found. Only a trend in the CG could be detected between low beta band and the SEE subscale “Lack of Emotions” (EO: *r* = 0.346, *P* = .066), indicating that increases in “Lack of Emotions” from before to after intervention could be related to increases in low beta band from before to after TAU in the CG (but would not withstand a correction for multiple testing).

Regarding changes of the exploratory neurophysiological measures, a relationship between the trend of the alpha band decrease and the increase in the SEE-subscale “Emotional Imaginary” was found in the EO condition (EO: *r* = −0.301, *P* = .024). Post-hoc tests confirmed this trend finding, i.e. a trend in the EG (EO: *r* = −0.360, *P* = .065), and further revealed a trend for a link between the trend of the alpha band decrease and the increase in the SEE-subscale “Impulsive Drinking Behavior” (EO: *r* = 0.352, *P* = .072) in the EG, while no significant interactions were found in the CG.

Exploratory analysis regarding relationships between rsEEG theta frequency band and the clinical symptomatology did not yield significant results.

## Discussion

Based on previous successful protocols (Peniston and Kulkosky 1989, 1991, 1999; Scott and Kaiser 1998; Scott et al. 2005), in the present study, clinical effects of an optimized combination of SMR and AT NF training were investigated in a population of adult patients with AUD.

Results on the clinical**–**psychological level in the affective domain reveal improvements in the main outcome, namely emotional competences, demonstrating increases in “Emotion Regulation,” while the “Lack of Emotions” was found to be improved, i.e. decreased after the experimental NF training. Although the SEE questionnaire is said to reflect more enduring personality dimensions (File et al. 2014), we found significant changes in those affective domains, symbolizing changes in emotional competence. Considering the construct “Emotion Regulation” as the most reported underlying and/or mediating factor between many other emotional competences and abilities (Brasseur et al. 2013, Mikolajczak et al. 2015, Naragon-Gainey et al. 2017, Schoon 2021), which is at the same time severely impaired in patients with AUD, an increase in emotion regulation in those patients may enable other domains of emotional abilities in a similar vein to renew and strengthen. Finally, the description of an emotional deafness, numbness, and emptiness is one of the most often reported (un)emotional states in AUD (Khosravani et al. 2017, Bradizza et al. 2018). The decrease in “Lack of Emotions” found in this study may indicate the restoring and reinitiating of basic emotional intelligence, i.e. enabling the perception of emotions to facilitate a more developed emotion consciousness.

In general, an improved and restored emotional intelligence (i.e. emotional competence), leading from the perception and awareness of emotion to emotion recognition, empathic processes, emotional reflection, and self-efficacy to the ability to create connections and self-control, regulate and manage emotions of one self and others, is highly important, especially for patients with AUD. Deficits in emotion regulation processes can have a powerful impact on drinking behavior and relapse in AUD (Fox et al. 2008, Mohagheghi et al. 2015, Petit et al. 2015), so improving emotional competences by including EEG NF training in the treatment of patients with AUD might therefore prevent in a further step also from high relapse rates.

Although a trend regarding a group difference in “Impulsive Drinking Behavior” between the EG and CG was found after the interventions, valid changes, i.e. strong improvements concerning drinking behavior and alcohol-related cognitions, could not be confirmed by the results of this study. Rather, it seems that time and the conducted established ARP affects AUD symptoms on the cognitive-behavioral level in the same vein than with the additional NF training.

The exploratory analysis of this study regarding changes in further clinical**–**psychological symptoms spotted a multiplicity of improvements. Firstly, one of the main psychological factors influencing almost all psychopathological symptoms, namely the subjective quality of life, was found to be improved, i.e. life satisfaction regarding health as well as personal aspects of life satisfaction were observed to be increased after the experimental NF training. The observed improvement in such an established general mental health indicator—life satisfaction—strengthens the evidence toward positive health effects of NF training (Dehghani-Arani et al. 2013; Gruzelier 2014a, 2014b). In the same vein, cognitive coping strategies, as well as the ability to premediate and to persevere, also improved in patients with AUD after NF training.

Our analysis on the neurophysiological level expanded these findings, indicating centroparietal decreases in low beta band power, as well as a trend regarding a decrease in centroparietal alpha band power from preintervention to postintervention in the EG, compared to the CG. Considering a desynchronization, potentially in the form of an observed suppression of Rolandic beta rhythm, the accompanying decrease of EEG power in alpha frequency range, a potential mu-rhythm desynchronization is comprehensible. Partly similarly, Arns et al. (2012) found a decrease in SMR power (like low beta band power) post-treatment. On the contrary, Kober and colleagues (Kober et al. 2017) reported a linear increase in SMR band and concomitant slower frequencies during training and assumed non-EEG-band-specific effects through SMR band training.

Although core domains of emotional competence, such as emotion regulation processes, were repeatedly reported to be linked to centroparietal regions (Morawetz et al. 2016, Dominguez-Centeno et al. 2020, Hua et al. 2020), our study could not confirm any relationships between the reported improvements in emotion regulation and the lack of emotions and the reported decline in centroparietal low beta band power. The trend regarding the potential relationship between increases in low beta frequency band and increases in the lack of emotions in the CG could point toward positive effects regarding the decrease in low beta frequency band after the NF training of the EG, but it needs to be, similar to the above-mentioned brain-emotion/behavior link, investigated in more detail in future studies.

Taken together, the decrease in low beta power post-treatment might be explained by the hypothesis that NF enables voluntary control over a specific frequency, rather than alter the same frequency in rest (i.e. band power in rsEEG). This is in line with conclusions that the regulation of a specific EEG frequency over a specific EEG electrode could lead to the non-specific “entrainment” effect (Collura 2014), i.e. a general change of the EEG power of other nearby frequency bands and the cerebral cortex (Cheng et al. 2015, Gong et al. 2020, Kober et al. 2020).

Although it is not clear if outcomes are a synergetic effect of both NF protocols and reflect a mediating phenomenon in balancing and slowing down increased beta band brain activity in AUD, our results assuredly demonstrate significant improvements in the affective core symptoms of AUD in the EG compare to the CG. Although, our findings indicate that even a standard alcohol rehabilitation treatment, as our CG, shows effective improvements in reducing impulsive drinking behavior and alcohol-related cognition, the relationships between the neurophysiological level and the affective-behavioral-cognitive level still remain unclear. However, considering the improvements in core symptoms of AUD on the affective level, namely emotional competences, together with the observed balancing effect on the often reported, potentially psychopathologically increased low beta frequency band in AUD, our study broadens the path for implementing NF in already established alcohol-rehabilitation programs for improved recovery.

## Limitations

Almost all participants in the EG and CG (except one person in each group) of our study had at least one comorbid psychiatric disorder (e.g. nicotine drug dependence, cannabis use disorder, affective disorders, anxiety disorders, PTSD, or/and personality disorders), which presents a very heterogeneous study population. This is in accordance with research findings assuming individuals with AUD had one or more co-occurring comorbidities, especially anxiety disorder, mood disorder, and personality disorders (Grant et al. 2016, Yang et al. 2018). Therefore, the reported, limiting baseline differences in rsEEG between our EGs could eventually reflect the heterogeneous co-occurring psychiatric disorders in patients with AUD (Newson and Thiagarajan 2019, Choi et al. 2021, Liu et al. 2022), linked to different, additional pharmacological treatments (Iosifescu 2011, Haaf et al. 2023). An overview over the number of comorbid psychiatric disorder is provided in the Supplementary material (B) “Detailed Description of Clinical Sample.”

Another limitation of this study could be clearly seen in the special NF protocol sequence. The sequence of the NF parts (first: SMR, second: AT) could produce confounding findings, not enabling to disentangle the effects of each NF part alone or explore the effects of the reverse sequence (firstly AT, secondly: SMR). Therefore, we could only hypnotize if both NF training parts had synergetic effects on the reported self-reports on the clinical**–**psychological level and acted as two indispensable conditions or if one part had a greater impact on the measured indicators. Besides the stratification of comorbid disorders and related medication, as well as the inclusion of female patients with AUD, future research should further investigate different forms of neurotherapies, including NF, as well as approaches of neuromodulation, such as transcranial magnetic stimulation or transcranial direct current stimulation (Bari et al. 2018).

All procedures were in accordance with the ethical standards of the responsible committee on human experimentation (institutional and national) and with the Declaration of Helsinki 1975, as revised in 2000(5). Informed consent was obtained from all patients for being included in the study.

## Supplementary Material

nsae048_Supp

## References

[R1] Anton RF, Moak DH, Latham PK. The obsessive compulsive drinking scale: a new method of assessing outcome in alcoholism treatment studies. *Arch Gen Psychiatry* 1996;53:225–31. 8611059 10.1001/archpsyc.1996.01830030047008

[R2] Arns M, De Ridder S, Strehl U et al. Efficacy of neurofeedback treatment in ADHD: the effects on inattention, impulsivity and hyperactivity: a meta-analysis. *Clin EEG Neurosci* 2009;40:180–89. 19715181 10.1177/155005940904000311

[R3] Arns M, Drinkenburg W, Leon Kenemans J. The effects of QEEG-informed neurofeedback in ADHD: an open-label pilot study. *Appl Psychophysiol Biofeedback* 2012;37:171–80. 22446998 10.1007/s10484-012-9191-4 PMC3419351

[R4] Arns M, Feddema I, Kenemans JL. Differential effects of theta/beta and SMR neurofeedback in ADHD on sleep onset latency. *Front Human Neurosci* 2014;8:1019. 10.3389/fnhum.2014.01019 PMC4274876 25566034

[R5] Bari A, DiCesare J, Babayan D et al. Neuromodulation for substance addiction in human subjects: a review. *Neurosci Biobehav Rev* 2018;95:33–43. 30268433 10.1016/j.neubiorev.2018.09.013 PMC7405948

[R6] Bates ME, Buckman JF, Nguyen TT. A role for cognitive rehabilitation in increasing the effectiveness of treatment for alcohol use disorders. *Neuropsychol Rev* 2013;23:27–47. 23412885 10.1007/s11065-013-9228-3 PMC3610413

[R7] Beck AT, Steer RA, Brown G. *Beck Depression Inventory Manual*, 2nd edn. San Antonio, TX: The Psychological Corporation, 1996.

[R8] Behr M, Becker M (eds). *SEE-Skalen Zum Erleben von Emotionen*. Göttingen: Hogrefe, 2004.

[R9] Bell CC . DSM-IV: Diagnostic and Statistical Manual of Mental disorders. *JAMA* 1994;272:828–29.

[R10] Benjamini Y, Hochberg Y. Controlling the false discovery rate: a practical and powerful approach to multiple testing. *J R Stat Soc (Methodological)* 1995;57:289–300.

[R11] Berking M, Margraf M, Ebert D et al. Deficits in emotion-regulation skills predict alcohol use during and after cognitive–behavioral therapy for alcohol dependence. *J Consult Clin Psychol* 2011;79:307. 10.1037/a0023421 PMC3109184 21534653

[R12] Blanca MJ, Arnau J, García-Castro FJ et al. Non-normal data in repeated measures ANOVA: impact on type I error and power. *Psicothema* 2023;35:21–29. 36695847 10.7334/psicothema2022.292

[R13] Bradizza CM, Brown WC, Ruszczyk MU et al. Difficulties in emotion regulation in treatment-seeking alcoholics with and without co-occurring mood and anxiety disorders. *Addict Behav* 2018;80:6–13. 29306117 10.1016/j.addbeh.2017.12.033 PMC5807148

[R14] Brain Vision Analyzer 2.2 . Brain vision analyzer 2.2. Munich, Germany: BVA, 2006.

[R15] Brasseur S, Grégoire J, Bourdu R et al. The profile of emotional competence (PEC): development and validation of a self-reported measure that fits dimensions of emotional competence theory. *PLoS ONE* 2013;8:e62635. 10.1371/journal.pone.0062635 PMC3646043 23671616

[R16] Burkett VS, Cummins JM, Dickson RM et al. An open clinical trial utilizing real-time EEG operant conditioning as an adjunctive therapy in the treatment of crack cocaine dependence. *J Neurother* 2005;9:27–47.

[R17] Callaway TG, Bodenhamer-Davis E. Long-term follow-up of a clinical replication of the peniston protocol for chemical dependency. *J Neurother* 2008;12:243–59. 10.1080/10874200802502060

[R18] Campanella S, Petit G, Maurage P et al. Chronic alcoholism: insights from neurophysiology. *Neurophysiol Clin* 2009;39:191–207. 19853791 10.1016/j.neucli.2009.08.002

[R19] Cardenas VA, Studholme C, Gazdzinski S et al. Deformation-based morphometry of brain changes in alcohol dependence and abstinence. *Neuroimage* 2007;34:879–87. 17127079 10.1016/j.neuroimage.2006.10.015 PMC1865510

[R20] Cheng M-Y, Huang C-J, Chang Y-K et al. Sensorimotor rhythm neurofeedback enhances golf putting performance. *J Sport Exercise Psychol* 2015;37:626–36. 10.1123/jsep.2015-0166 26866770

[R21] Choi K-M, Kim J-Y, Kim Y-W et al. Comparative analysis of default mode networks in major psychiatric disorders using resting-state EEG. *Sci Rep* 2021;11:22007. 10.1038/s41598-021-00975-3 PMC8580995 34759276

[R22] Cohen J . *Statistical Power Analysis for the Behavioral Sciences*. Cambridge: Academic Press, 2013.

[R23] Collura TF . *Technical Foundations of Neurofeedback*. Routledge: Taylor & Francis Group, 2014.

[R24] Cui C, Noronha A, Warren KR et al. Brain pathways to recovery from alcohol dependence. *Alcohol* 2015;49:435–52. 26074423 10.1016/j.alcohol.2015.04.006 PMC4468789

[R25] Czapla M, Simon JJ, Richter B et al. The impact of cognitive impairment and impulsivity on relapse of alcohol‐dependent patients: implications for psychotherapeutic treatment. *Addict Biol* 2016;21:873–84. 25678237 10.1111/adb.12229

[R26] Dalkner N, Unterrainer HF, Wood G et al. Short-term beneficial effects of 12 sessions of neurofeedback on avoidant personality accentuation in the treatment of alcohol use disorder. *Front Psychol* 2017;8:1688. 10.3389/fpsyg.2017.01688 PMC5622970 29018397

[R27] Dehghani-Arani F, Rostami R, Nadali H. Neurofeedback training for opiate addiction: improvement of mental health and craving. *Appl Psychophysiol Biofeedback* 2013;38:133–41. 10.1007/s10484-013-9218-5 23605225 PMC3650238

[R28] Dominguez-Centeno I, Jurado-Barba R, Sion A et al. Psychophysiological correlates of emotional-and alcohol-related cues processing in offspring of alcohol-dependent patients. *Alcohol Alcohol* 2020;55:374–81. 32300797 10.1093/alcalc/agaa006

[R29] Doppelmayr M, Weber E. Effects of SMR and theta/beta neurofeedback on reaction times, spatial abilities, and creativity. *J Neurother* 2011;15:115–29.

[R30] Dousset C, Kajosch H, Ingels A et al. Preventing relapse in alcohol disorder with EEG-neurofeedback as a neuromodulation technique: a review and new insights regarding its application. *Addict Behav* 2020;106:106391. 10.1016/j.addbeh.2020.106391 32197211

[R31] Dvorak RD, Sargent EM, Kilwein TM et al. Alcohol use and alcohol-related consequences: associations with emotion regulation difficulties. *Am J Drug Alcohol Abuse* 2014;40:125–30. 24588419 10.3109/00952990.2013.877920

[R32] Egner T, Gruzelier JH. The temporal dynamics of electroencephalographic responses to alpha/theta neurofeedback training in healthy subjects. *J Neurother* 2004;8:43–57. 10.1300/J184v08n01_04

[R33] Egner T, Strawson E, Gruzelier JH. EEG signature and phenomenology of alpha/theta neurofeedback training versus mock feedback. *Appl Psychophysiol Biofeedback* 2002;27:261–70. 12557453 10.1023/a:1021063416558

[R34] Ehlers CL, Phillips E. EEG low‐voltage alpha and alpha power in African American young adults: relation to family history of alcoholism. *Alcohol Clin Exp Res* 2003;27:765–72. 12766620 10.1097/01.ALC.0000065439.09492.67

[R35] Enoch MA, Albaugh BJ. Genetic and environmental risk factors for alcohol use disorders in American Indians and Alaskan natives. *Am J Addict* 2017;26:461–68. 27599369 10.1111/ajad.12420 PMC5339067

[R36] Enriquez-Geppert S, Smit D, Pimenta MG et al. Neurofeedback as a treatment intervention in ADHD: current evidence and practice. *Curr Psychiatry Rep* 2019;21:1–7. 31139966 10.1007/s11920-019-1021-4 PMC6538574

[R37] Erdmann G, Janke W. Stressverarbeitungsfragebogen: SVF; Stress, Stressverarbeitung und ihre Erfassung durch ein mehrdimensionales Testsystem. Hogrefe, 2008.

[R38] Fahrenberg J, Myrtek M, Schumacher J et al. FLZ. *Fragebogen zur Lebenszufriedenheit. Göttingen*: Hogrefe. 2000.

[R39] Faul F, Erdfelder E, Lang A-G et al. G* Power 3: a flexible statistical power analysis program for the social, behavioral, and biomedical sciences. *Behav Res Methods* 2007;39:175–91. 17695343 10.3758/bf03193146

[R40] Fielenbach S, Donkers FC, Spreen M et al. Neurofeedback as a treatment for impulsivity in a forensic psychiatric population with substance use disorder: study protocol of a randomized controlled trial combined with an N-of-1 clinical trial. *JMIR Res Protoc* 2017;6:e13. 10.2196/resprot.6907 PMC5299210 28122696

[R41] Fielenbach S, Donkers FC, Spreen M et al. Effects of a theta/sensorimotor rhythm neurofeedback training protocol on measures of impulsivity, drug craving, and substance abuse in forensic psychiatric patients with substance abuse: randomized controlled trial. *JMIR Ment Health* 2018;5:e10845. 10.2196/10845 PMC6305873 30538087

[R42] Fielenbach S, Donkers F, Spreen M et al. Theta/SMR neurofeedback training works well for some forensic psychiatric patients, but not for others: a sham-controlled clinical case series. *Int J Offender Ther Comp Criminol* 2019;63:2422–39. 31130043 10.1177/0306624X19849562

[R43] File N, Keil WW, Sauer J et al. Ansätze zur empirischen forschung in der klientenzentrierten psychotherapie in Österreich. *Person* 2014;18:18–30.

[R44] Fitzpatrick LE, Crowe SF. Cognitive and emotional deficits in chronic alcoholics: a role for the cerebellum? *Cerebellum* 2013;12:520–33. 23436003 10.1007/s12311-013-0461-3

[R45] Fox H, Hong K, Sinha R. Difficulties in emotion regulation and impulse control in recently abstinent alcoholics compared with social drinkers. *Addict Behav* 2008;33:388–94. 18023295 10.1016/j.addbeh.2007.10.002

[R46] Gadea M, Aliño M, Hidalgo V et al. Effects of a single session of SMR neurofeedback training on anxiety and cortisol levels. *Neurophysiologie Clin* 2020;50:167–73. 10.1016/j.neucli.2020.03.001 32279927

[R47] Garland EL . Mindful positive emotion regulation as a treatment for addiction: from hedonic pleasure to self-transcendent meaning. *Curr Opin Behav Sci* 2021;39:168–77. 34084873 10.1016/j.cobeha.2021.03.019 PMC8168946

[R48] Gilmore CS, Fein G. Theta event-related synchronization is a biomarker for a morbid effect of alcoholism on the brain that may partially resolve with extended abstinence. *Brain Behav* 2012;2:796–805. 10.1002/brb3.95 23170242 PMC3500466

[R49] Gong A, Nan W, Yin E et al. Efficacy, trainability, and neuroplasticity of SMR vs. alpha rhythm shooting performance neurofeedback training. *Front Hum Neurosci* 2020;14:94. 10.3389/fnhum.2020.00094 PMC7098988 32265676

[R50] Grant BF . ICD‐10 harmful use of alcohol and the alcohol dependence syndrome: prevalence and implications. *Addiction* 1993;88:413–20. 8461858 10.1111/j.1360-0443.1993.tb00829.x

[R51] Grant BF, Saha TD, Ruan WJ et al. Epidemiology of DSM-5 drug use disorder: results from the national epidemiologic survey on alcohol and related conditions–III. *JAMA Psychiatry* 2016;73:39–47. 26580136 10.1001/jamapsychiatry.2015.2132 PMC5062605

[R52] Gruzelier J . A theory of alpha/theta neurofeedback, creative performance enhancement, long distance functional connectivity and psychological integration. *Cogn Process* 2009;10:101–09. 19082646 10.1007/s10339-008-0248-5

[R53] Gruzelier J, Egner T, Vernon D. Validating the efficacy of neurofeedback for optimising performance. *Prog Brain Res* 2006;159:421–31. 17071246 10.1016/S0079-6123(06)59027-2

[R54] Gruzelier JH . Differential effects on mood of 12–15 (SMR) and 15–18 (beta1) Hz neurofeedback. *Int J Psychophysiol* 2014a;93:112–15. 23357178 10.1016/j.ijpsycho.2012.11.007

[R55] Gruzelier JH . EEG-neurofeedback for optimising performance. I: a review of cognitive and affective outcome in healthy participants. *Neurosci Biobehav Rev* 2014b;44:124–41. 24125857 10.1016/j.neubiorev.2013.09.015

[R56] Haaf M, Curic S, Rauh J et al. Opposite modulation of the NMDA receptor by glycine and S-ketamine and the effects on resting state EEG gamma activity: new insights into the glutamate hypothesis of schizophrenia. *Int J Mol Sci* 2023;24:1913. 10.3390/ijms24031913 PMC9916476 36768234

[R57] Herting MM, Fair D, Nagel BJ. Altered fronto-cerebellar connectivity in alcohol-naive youth with a family history of alcoholism. *Neuroimage* 2011;54:2582–89. 20970506 10.1016/j.neuroimage.2010.10.030 PMC3150517

[R58] Hoyer J . Der Fragebogen zur Dysfunktionalen und Funktionalen Selbstaufmerksamkeit (DFS): Theoretisches Konzept und Befunde zur Reliabilität und Validität. *Diagnostica* 2000;46:140–48.

[R59] Hua JP, Trull TJ, Merrill AM et al. Daily-life affective instability in emotional distress disorders is associated with function and structure of posterior parietal cortex. *Psychiatry Res Neuroim* 2020;296:111028. 10.1016/j.pscychresns.2019.111028 31911320

[R60] Huang Y, Mohan A, De Ridder D et al. The neural correlates of the unified percept of alcohol-related craving: a fMRI and EEG study. *Sci Rep* 2018;8:923. 10.1038/s41598-017-18471-y PMC5772563 29343732

[R61] IBM SPSS Statistics 27 . *IBM SPSS Statistics 27, IBM*. New York: IBM Company Armonk, 2011.

[R62] Iosifescu DV . Electroencephalography-derived biomarkers of antidepressant response. *Harv Rev Psychiatry* 2011;19:144–54. 21631160 10.3109/10673229.2011.586549

[R63] Jakubczyk A, Trucco EM, Kopera M et al. The association between impulsivity, emotion regulation, and symptoms of alcohol use disorder. *J Subst Abuse Treat* 2018;91:49–56. 29910014 10.1016/j.jsat.2018.05.004 PMC6020846

[R64] Jason B, Ye Y, Cherpitel CJ et al. Scaling properties of the combined ICD-10 dependence and harms criteria and comparisons with DSM-5 alcohol use disorder criteria among patients in the emergency department. *J Stud Alcohol Drugs* 2012;73:328–36. 22333341 10.15288/jsad.2012.73.328 PMC3281989

[R65] Jasper HH . Report of the committee on methods of clinical examination in electroencephalography: 1957. *Electroencephalogr Clin Neurophysiol* 1958;10:370–75.

[R66] Julien J, Ayer T, Tapper EB et al. Effect of increased alcohol consumption during COVID‐19 pandemic on alcohol‐associated liver disease: a modeling study. *Hepatology* 2022;75:1480–90. 34878683 10.1002/hep.32272 PMC9015640

[R67] Kamarajan C . Brain electrophysiological signatures in human alcoholism and risk. In The *Neuroscience of Alcohol*. pp. 119–30. San Diego: Elsevier, Academic Press, 2019.

[R68] Khantzian EJ . Self-regulation and self-medication factors in alcoholism and the addictions. Similarities and differences. *Recent Dev Alcohol* 1990;8:255–71. 2185521

[R69] Khosravani V, Bastan FS, Ghorbani F et al. Difficulties in emotion regulation mediate negative and positive affects and craving in alcoholic patients. *Addict Behav* 2017;71:75–81. 28273489 10.1016/j.addbeh.2017.02.029

[R70] Kober H, Bolling D, (eds). *Emotion Regulation in Substance Use Disorders*, Vol. 2, New York: Guilford Press, 2014.

[R71] Kober SE, Neuper C, Wood G. Differential effects of up-and down-regulation of SMR coherence on EEG activity and memory performance: a neurofeedback training study. *Front Human Neurosci* 2020;14:606684. 10.3389/fnhum.2020.606684 PMC7793696 33424569

[R72] Kober SE, Witte M, Neuper C et al. Specific or nonspecific? Evaluation of band, baseline, and cognitive specificity of sensorimotor rhythm-and gamma-based neurofeedback. *Int J Psychophysiol* 2017;120:1–13. 28652143 10.1016/j.ijpsycho.2017.06.005

[R73] Kober SE, Witte M, Stangl M et al. Shutting down sensorimotor interference unblocks the networks for stimulus processing: An SMR neurofeedback training study. *Clin Neurophysiol* 2015;126:82–95. 24794517 10.1016/j.clinph.2014.03.031

[R74] Konicar L, Veit R, Eisenbarth H et al. Brain self-regulation in criminal psychopaths. *Sci Rep* 2015;5:1–7. 10.1038/srep09426 PMC4371087 25800672

[R75] Lackner N, Unterrainer HF, Skliris D et al. The effectiveness of visual short-time neurofeedback on brain activity and clinical characteristics in alcohol use disorders: practical issues and results. *Clin EEG and Neurosci* 2016;47:188–95. 10.1177/1550059415605686 26415612

[R76] Lejuez CW, Magidson JF, Mitchell SH et al. Behavioral and biological indicators of impulsivity in the development of alcohol use, problems, and disorders. *Alcohol Clin Exp Res* 2010;34:1334–45. 20491733 10.1111/j.1530-0277.2010.01217.x PMC3182265

[R77] Liu Y, Chen Y, Fraga-González G et al. Resting-state EEG, substance use and abstinence after chronic use: a systematic review. *Clin EEG and Neurosci* 2022;53:344–66. 10.1177/15500594221076347 35142589

[R78] Marzbani H, Marateb HR, Mansourian M. Neurofeedback: a comprehensive review on system design, methodology and clinical applications. *Basic Clin Neurosci* 2016;7:143–58. 10.15412/J.BCN.03070208 27303609 PMC4892319

[R79] Mathews BL, Koehn AJ, Abtahi MM et al. Emotional competence and anxiety in childhood and adolescence: A meta-analytic review. *Clin Child Fam Psychol Rev* 2016;19:162–84. 27072682 10.1007/s10567-016-0204-3

[R80] Mayer JD, Salovey P, Caruso D. Models of emotional intelligence. *Handbook Intell* 2000;2:396–420.

[R81] Mikolajczak M, Avalosse H, Vancorenland S et al. A nationally representative study of emotional competence and health. *Emotion* 2015;15:653. 10.1037/emo0000034 25893449

[R82] Milnik V, Buchner H, Blankenstein J. Das 10–20-Elektrodensystem–praktisch. *Klin Neurophysiol* 2020;51:242–44.

[R83] Mohagheghi A, Amiri S, Mousavi Rizi S et al. Emotional intelligence components in alcohol dependent and mentally healthy individuals. *Sci World J* 2015;2015:841039. 10.1155/2015/841039 PMC4393918 25893214

[R84] Morawetz C, Bode S, Baudewig J et al. Neural representation of emotion regulation goals. *Human Brain Mapp* 2016;37:600–20. 10.1002/hbm.23053 PMC6867353 26537018

[R85] Mumtaz W, Vuong PL, Malik AS et al. A review on EEG-based methods for screening and diagnosing alcohol use disorder. *Cogn Neurodyn* 2018;12:141–56. 29564024 10.1007/s11571-017-9465-x PMC5852012

[R86] Musalek M . Ressource-oriented treatment of addiction-the orpheus programme. *Eur Psychiatry* 2011;26:2017.

[R87] Naragon-Gainey K, McMahon TP, Chacko TP. The structure of common emotion regulation strategies: a meta-analytic examination. *Psychol Bull* 2017;143:384. 10.1037/bul0000093 28301202

[R88] Newson JJ, Thiagarajan TC. EEG frequency bands in psychiatric disorders: a review of resting state studies. *Front Human Neurosci* 2019;12:521. 10.3389/fnhum.2018.00521 PMC6333694 30687041

[R89] Niv S . Clinical efficacy and potential mechanisms of neurofeedback. *Pers Individ Dif* 2013;54:676–86. 10.1016/j.paid.2012.11.037

[R90] O’Brien C . Addiction and dependence in DSM‐V. *Addiction* 2011;106:866–67. 21477226 10.1111/j.1360-0443.2010.03144.x PMC3812919

[R91] O’daly OG, Trick L, Scaife J et al. Withdrawal-associated increases and decreases in functional neural connectivity associated with altered emotional regulation in alcoholism. *Neuropsychopharmacology* 2012;37:2267–76. 22617355 10.1038/npp.2012.77 PMC3422491

[R92] OECD . Preventing harmful alcohol. In: Studies HP (ed), Paris: OECD Publishing, 2021.

[R93] Ottonello M, Fiabane E, Pistarini C et al. Difficulties in emotion regulation during rehabilitation for alcohol addiction: correlations with metacognitive beliefs about alcohol use and relapse risk. *Neuropsychiatr Dis Treat* 2019;15:2917–25. 31686826 10.2147/NDT.S214268 PMC6798816

[R94] Peniston EG, Kulkosky PJ. α‐θ Brainwave training and β‐endorphin levels in alcoholics. *Alcohol Clin Exp Res* 1989;13:271–79. 2524976 10.1111/j.1530-0277.1989.tb00325.x

[R95] Peniston EG, Kulkosky PJ. Alpha-theta brainwave neurofeedback for Vietnam veterans with combat-related post-traumatic stress disorder. *Med Psychother* 1991;4:47–60.

[R96] Peniston EG, Kulkosky PJ. Neurofeedback in the treatment of addictive disorders. In: *Introduction to Quantitative EEG and Neurofeedback*. pp. 157–79. San Diego: Elsevier, Academic Press, 1999.

[R97] Petit G, Luminet O, Maurage F et al. Emotion regulation in alcohol dependence. *Alcohol Clin Exp Res* 2015;39:2471–79. 26613633 10.1111/acer.12914

[R98] Porjesz B, Rangaswamy M, Kamarajan C et al. The utility of neurophysiological markers in the study of alcoholism. *Clin Neurophysiol* 2005;116:993–1018. 15826840 10.1016/j.clinph.2004.12.016

[R99] Rangaswamy M, Porjesz B. Understanding alcohol use disorders with neuroelectrophysiology. *Handbook Clin Neurol* 2014;125:383–414. 10.1016/B978-0-444-62619-6.00023-9 PMC4331067 25307587

[R100] Rangaswamy M, Porjesz B, Chorlian DB et al. Beta power in the EEG of alcoholics. *Biol. Psychiatry* 2002;52:831–42. 12372655 10.1016/s0006-3223(02)01362-8

[R101] Rangaswamy M, Porjesz B, Chorlian DB et al. Resting EEG in offspring of male alcoholics: beta frequencies. *Int J Psychophysiol* 2004;51:239–51. 10.1016/j.ijpsycho.2003.09.003 14962576

[R102] Rapaport MH, Tipp JE, Schuckit MA. A comparison of ICD-10 and DSM-III-R criteria for substance abuse and dependence. *Am J Drug Alcohol Abuse* 1993;19:143–51. 8387239 10.3109/00952999309002675

[R103] Rawls E, Kummerfeld E, Zilverstand A. An integrated multimodal model of alcohol use disorder generated by data-driven causal discovery analysis. *Commun Biol* 2021;4:435. 10.1038/s42003-021-01955-z PMC8012376 33790384

[R104] Riley H, Schutte NS. Low emotional intelligence as a predictor of substance-use problems. *J Drug Educ* 2003;33:391–98. 15237864 10.2190/6DH9-YT0M-FT99-2X05

[R105] Ros T, Enriquez-Geppert S, Zotev V et al. *Consensus on the Reporting and Experimental Design of Clinical and Cognitive-behavioural Neurofeedback Studies (CRED-nf Checklist*). Brain, 2020, 6 (1674-1685), Oxford University Press. 10.1093/brain/awaa009 PMC7296848 32176800

[R106] Rostami R, Dehghani-Arani F. Neurofeedback training as a new method in treatment of crystal methamphetamine dependent patients: a preliminary study. *Appl Psychophysiol Biofeedback* 2015;40:151–61. 25894106 10.1007/s10484-015-9281-1

[R107] Russo GM, Smith S, Sperandio KR. A meta‐analysis of neurofeedback for treating substance use disorders. *J Couns Dev* 2023;101:143–56.

[R108] Saarni C . *The Development of Emotional Competence*. New York: Guilford Press, 1999.

[R109] Sallie SN, Ritou V, Bowden-Jones H et al. Assessing international alcohol consumption patterns during isolation from the COVID-19 pandemic using an online survey: highlighting negative emotionality mechanisms. *BMJ Open* 2020;10:e044276. 10.1136/bmjopen-2020-044276 PMC7692002 33243820

[R110] Saunders JB, Degenhardt L, Reed GM et al. Alcohol use disorders in ICD‐11: Past, present, and future. *Alcohol Clin Exp Res* 2019;43:1617–31. 31194891 10.1111/acer.14128

[R111] Saxby E, Peniston EG. Alpha‐theta brainwave neurofeedback training: An effective treatment for male and female alcoholics with depressive symptoms. *J Clin Psychol* 1995;51:685–93. 8801245 10.1002/1097-4679(199509)51:5<685::aid-jclp2270510514>3.0.co;2-k

[R112] Schepis TS, Rao U, Yadav H et al. The limbic–hypothalamic–pituitary–adrenal axis and the development of alcohol use disorders in youth. *Alcohol Clin Exp Res* 2011;35:595–605. 21223300 10.1111/j.1530-0277.2010.01380.x PMC3074933

[R113] Schmidt RE, Gay P, d’Acremont M et al. A German adaptation of the UPPS impulsive behavior scale: psychometric properties and factor structure. *Swiss J Psychol* 2008;67:107–12.

[R114] Schoon I . Towards an integrative taxonomy of social-emotional competences. *Front Psychol* 2021;12:515313. 10.3389/fpsyg.2021.515313 PMC8005751 33790819

[R115] Scott WC, Kaiser D, Othmer S et al. Effects of an EEG biofeedback protocol on a mixed substance abusing population. *Am J Drug Alcohol Abuse* 2005;31:455–69. 10.1081/ada-200056807 16161729

[R116] Scott W, Kaiser D. Augmenting chemical dependency treatment with neurofeedback training. *J Neurother* 1998;3:66.

[R117] Sokhadze TM, Cannon RL, Trudeau DL. EEG biofeedback as a treatment for substance use disorders: review, rating of efficacy, and recommendations for further research. *Appl Psychophysiol Biofeedback* 2008;33:1–28. 10.1007/s10484-007-9047-5 18214670 PMC2259255

[R118] Stenberg G . Personality and the EEG: arousal and emotional arousability. *Pers Individ Dif* 1992;13:1097–113.

[R119] Van Doren J, Arns M, Heinrich H et al. Sustained effects of neurofeedback in ADHD: a systematic review and meta-analysis. *Eur Child Adolesc Psychiatry* 2019;28:293–305. 29445867 10.1007/s00787-018-1121-4 PMC6404655

[R120] Wang J, Fan Y, Dong Y et al. Combining gray matter volume in the cuneus and the cuneus-prefrontal connectivity may predict early relapse in abstinent alcohol-dependent patients. *PLoS One* 2018;13:e0196860. 10.1371/journal.pone.0196860 PMC5937790 29734343

[R121] Weissman DG, Schriber RA, Fassbender C et al. Earlier adolescent substance use onset predicts stronger connectivity between reward and cognitive control brain networks. *Dev Cogn Neurosci* 2015;16:121–29. 26215473 10.1016/j.dcn.2015.07.002 PMC4691372

[R122] Wetherill RR, Bava S, Thompson WK et al. Frontoparietal connectivity in substance-naive youth with and without a family history of alcoholism. *Brain Res* 2012;1432:66–73. 22138427 10.1016/j.brainres.2011.11.013 PMC3246051

[R123] White Nancy E . Theories of the effectiveness of alpha-theta training for multiple disorders. In: *Introduction to Quantitative EEG and Neurofeedback*. pp. 341–67. New York: Elsevier, Academic Press, 1999.

[R124] WHO . *The ICD-10 Classification of Mental and Behavioural Disorders: Clinical Descriptions and Diagnostic Guidelines*, Vol. 1. Geneva: World Health Organization, 1992.

[R125] WHO . Digital Marketing of Alcohol: Challenges and Policy Options for Better Health in the WHO European Region. In: Carlin E, Hellman M (ed.), Copenhagen: WHO regional office of europe, 2021.

[R126] Wilcox CE, Pommy JM, Adinoff B. Neural circuitry of impaired emotion regulation in substance use disorders. *Am J Psychiatry* 2016;173:344–61. 26771738 10.1176/appi.ajp.2015.15060710 PMC4979988

[R127] Witkiewitz K, Litten R, Leggio L. Advances in the science and treatment of alcohol use disorder. *Sci Adv* 2019;5:40-43. 10.1126/sciadv.aax4043 PMC6760932 31579824

[R128] Yang P, Tao R, He C et al. The risk factors of the alcohol use disorders-through review of its comorbidities. *Front Neurosci* 2018;12:303. 10.3389/fnins.2018.00303 PMC5958183 29867316

[R129] Yoshimura A, Komoto Y, Higuchi S. Exploration of core symptoms for the diagnosis of alcohol dependence in the ICD‐10. *Alcohol Clin Exp Res* 2016;40:2409–17. 27716976 10.1111/acer.13225 PMC5108416

