# Supplementary material for: Brain regulation training improves emotional competences in patients with alcohol use disorder
Source: Soc Cogn Affect Neurosci. 2024 Jun 25;19(1):nsae048. doi: 10.1093/scan/nsae048 (PMC11297497; doi:10.1093/scan/nsae048)
Supplement: nsae048_Supp [file nsae048_supp.zip › scan-23-223-File009.docx]

**Supplementary Information**

Brain regulation training improves

emotional competences

in patients with severe alcohol use disorder

Hack, R.L.^1,2^, Aigner, M.^3^, Musalek, M. ^2,^ Crevenna, R.^4^ & Konicar, L.^1^

**Contents**

| (A) | CRED-nf Checklist | 2 |
| --- | --- | --- |
| (B) | Detailed Description of Clinical Sample | 4 |
| (C) | Detailed Analysis of ‘Emotional Competence’ Total Score | 5 |
| (D) | Neurofeedback Learning & Regulation Strategies | 6 |
|  |  |  |

*
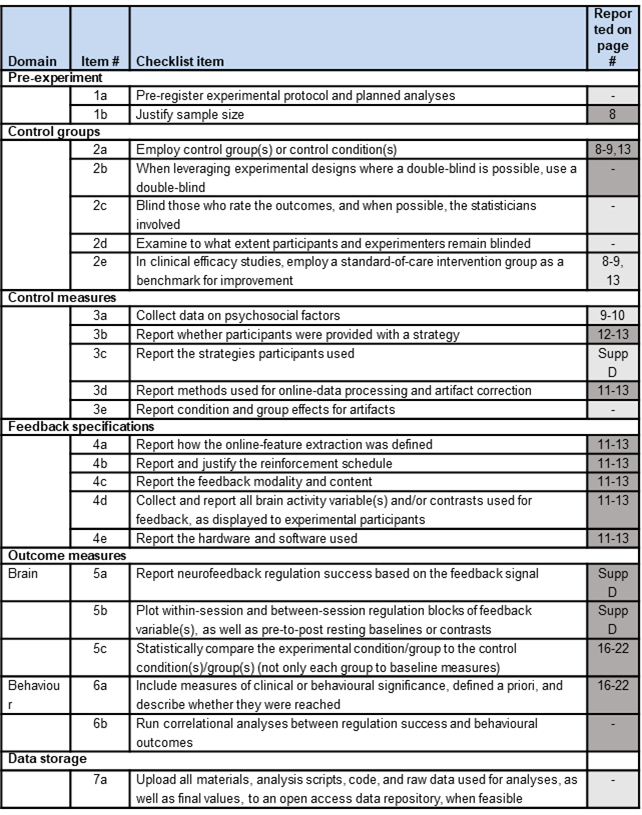
* **(A) Consensus on the Reporting and Experimental Design of clinical and cognitive-behavioral Neurofeedback studies (CRED-nf) best practices checklist 2020***

*Darker shaded boxes represent *Essential* checklist items; lightly shaded boxes represent *Encouraged* checklist items.

**(B) Detailed Description of Clinical Sample**

**(C) Detailed Analysis of ‘Emotional Competence’ Total Score**

For comparison reasons, we here additionally provide a repeated-measures analysis of variance with the Total Scale ‘*Emotional Competence’* of the SEE (Behr & Becker, 2004)) revealing a significant main effect of *‘Time’* (*F*(1,54)= 13.26; *p*< .001; *η²= .197*) with lower scores before interventions (T1) compared to after interventions (T2), as well as a significant interaction ‘*Time*Group’* (*F*(1,54)= 8.59; *p*< .005; *η²= .137*). Pairwise post-hoc tests revealed a significant increase in “*Emotion Competence*” (*t*_(26)_= -3.99, *p*< .001, *d*=-.77) from before (*m*= 48.58, *SD*= 19.91) to after intervention (*m*= 61.92, *SD*= 23.60) in the experimental group. No significant changes in ‘*Emotional Competence’* could be observed in the control group. No significant differences between the EG and the CG were found at baseline (T1).


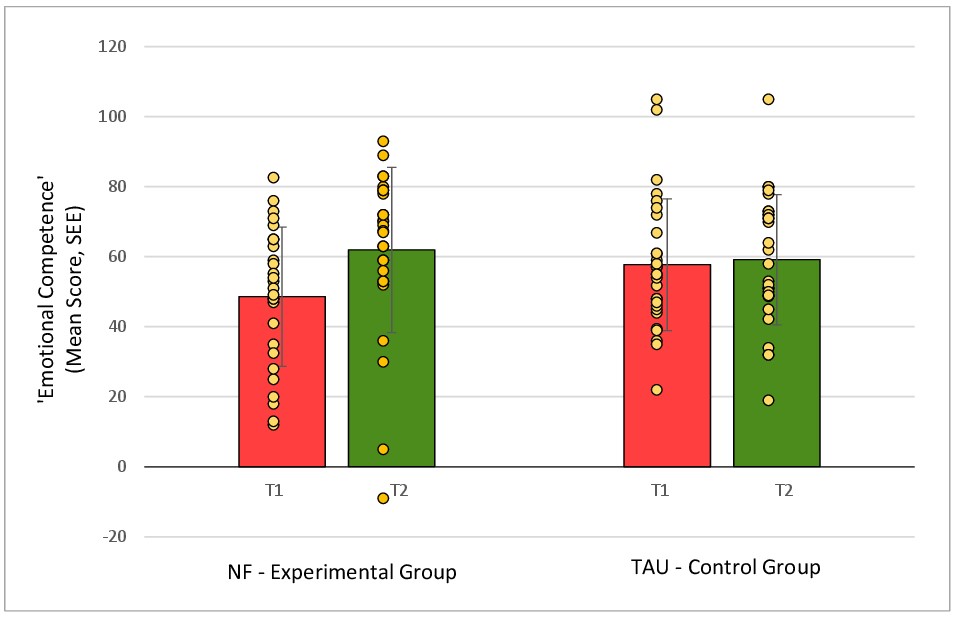


**Figure 3c: Increases in the ‘Emotional Competence’ (Total Score, SEE**: Scales for Experiencing Emotion (Behr & Becker, 2004)) from before intervention (T1, red bars) to after intervention (T2, green bars) of Neurofeedback (NF, *n*_NF_ = 27) - Experimental Group and Treatment As Usual (TAU, *n*_TAU_ = 29) Control Group

**(D) Neurofeedback Learning & Regulation Strategies**


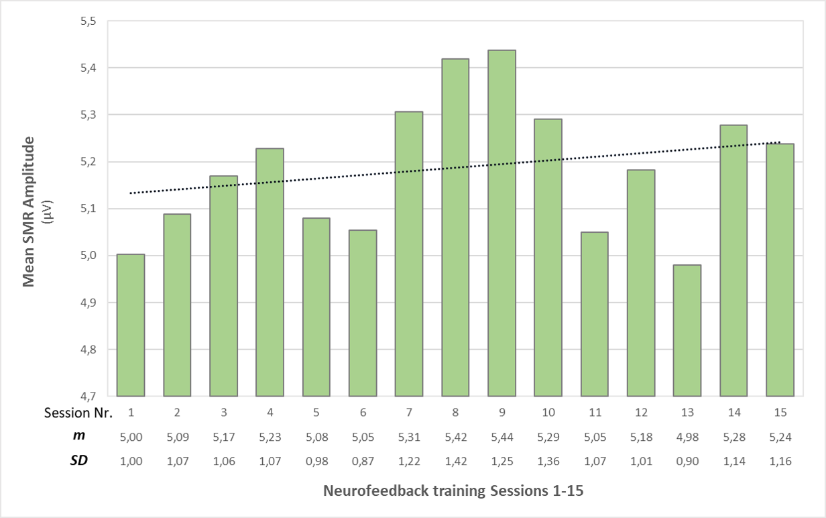

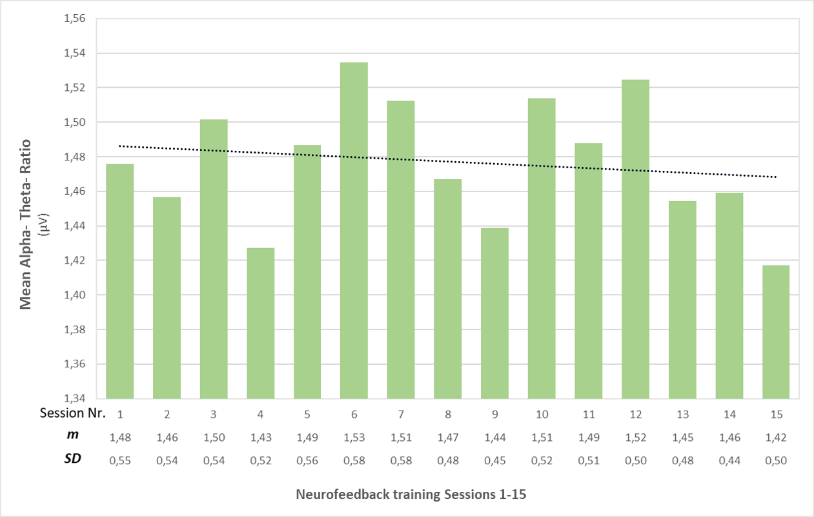
In addition to the focus on the primary outcome: emotional competences and on related changes in psychopathology and psychophysiology, we here provide the first basic neurofeedback learning results, based on the raw EEG data of the NeXus-32 channel Neuro-/Biofeedback System / BioTrace+Software (Mind Media; Herten, Netherlands). All 27 participants receiving the established alcohol rehabilitation program and the EEG-based brain training successfully completed the experimental neurofeedback training. The highest group mean peaks could be observed for SMR amplitude between neurofeedback session 7-9 and for alpha-theta-ratio (ATR) between session 6 and 7, and session 10 and 11, which indicates a slight increase in SMR regulation success over the progress of neurofeedback training, as well as a trend regarding an approximate approach to the desirable target size of an alpha/theta- ratio (ATR) close to 1. Fine-grained analysis regarding the course of neurofeedback training and the potential influence of the different neurofeedback training parts (SMR, ATR) will be addressed in detail in related, upcoming future investigations.

**Figure 5a: Mean SMR Amplitude**

**Figure 5b. Mean Alpha- Theta- Ratio**

Figure 5ab: Mean SMR Amplitude (Fig.5a) and Mean Alpha- Theta- Ratio (Fig.5b) for Neurofeedbacktraining Sessions 1-15 of the Experimental Group (n*EG*=27) including session means (m) and standard deviations (SD)

According to our qualitative neurofeedback strategy documentation, the used strategies for regulating the required brain activity could be roughly categorized into the following regulation domains of Haslinger et al. (2020): 1) theme ‘Mindfullness/ Relaxation’ of the cognitive domain (regulation strategy: e.g., mindfulness on waves) and 2) theme ’Generating Internal Phenomena’ of the cognitive domain (regulation strategy: e.g., imagination of words). Besides that, the majority of participants did not mention any regulation strategy or only reported to watch different display screens (regulation strategy: tracking of e.g. the a) SMR display bar, b) the muscle tension display bar or c) the score table on the client screen.
