# Supplementary figures and images for: Brain regulation training improves emotional competences in patients with alcohol use disorder
Source: Soc Cogn Affect Neurosci. 2024 Jun 25;19(1):nsae048. doi: 10.1093/scan/nsae048 (PMC11297497; doi:10.1093/scan/nsae048)

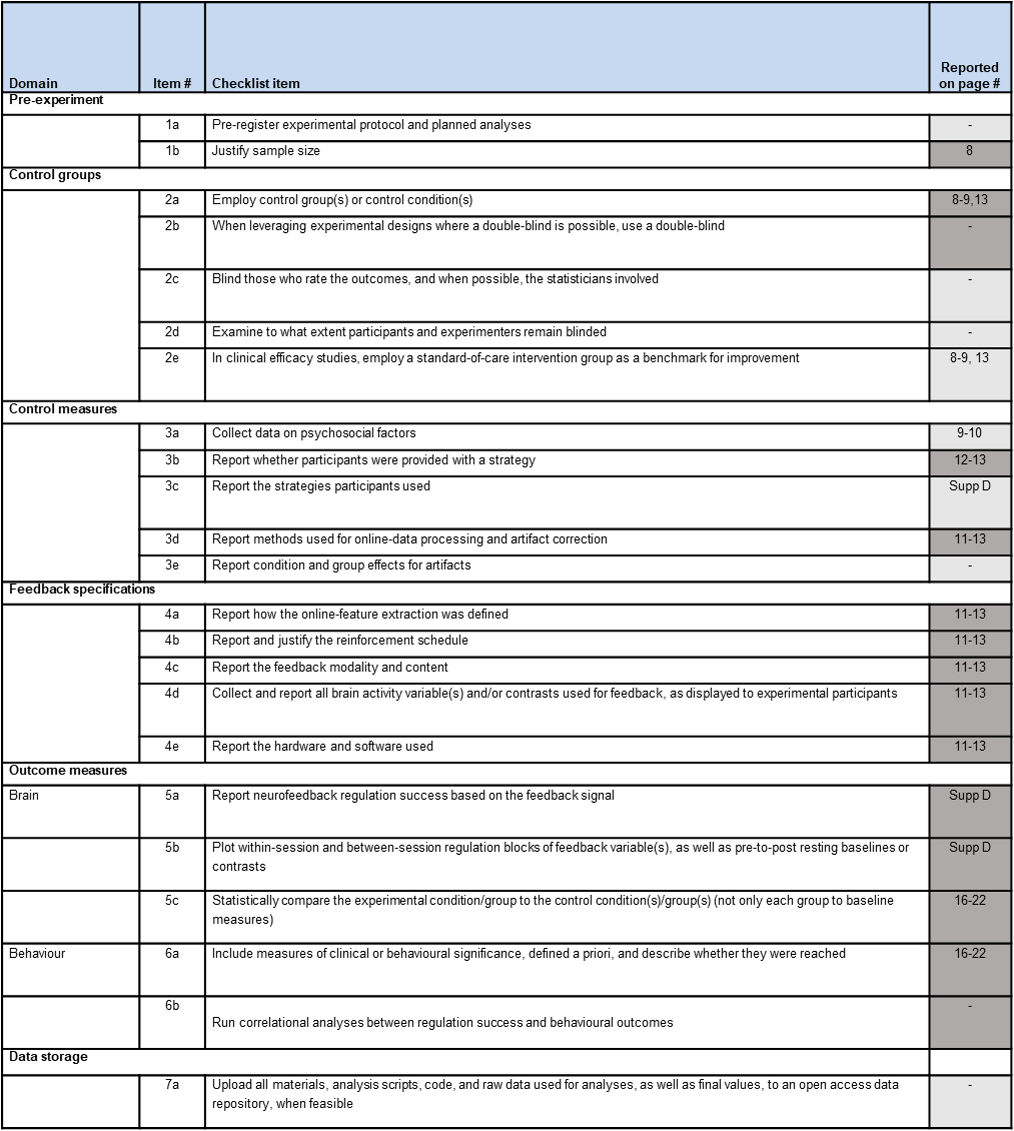

Supplement: nsae048_Supp [file nsae048_supp.zip › scan-23-223-File010.tif]

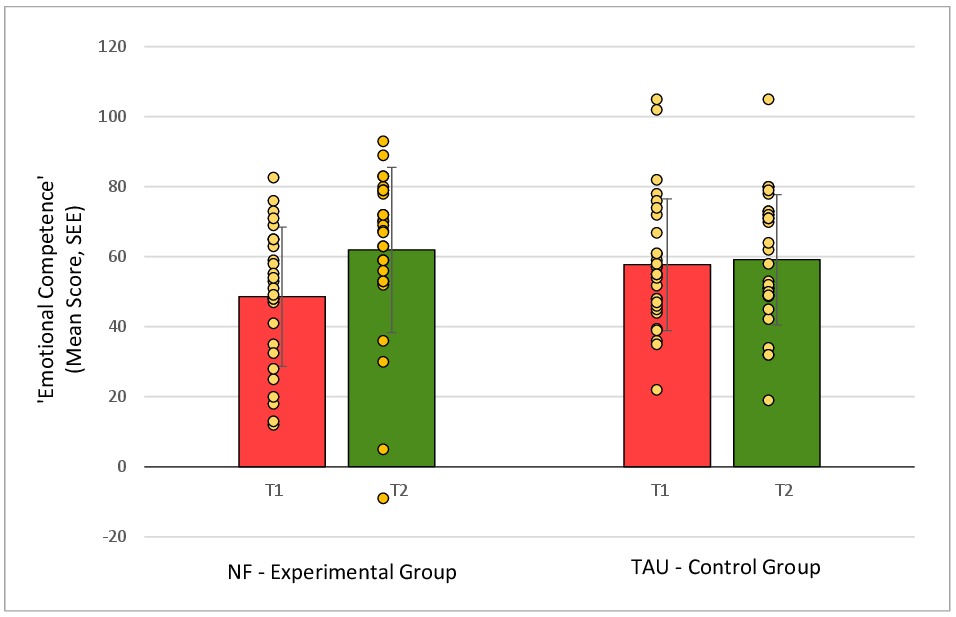

Supplement: nsae048_Supp [file nsae048_supp.zip › scan-23-223-File011.tiff]

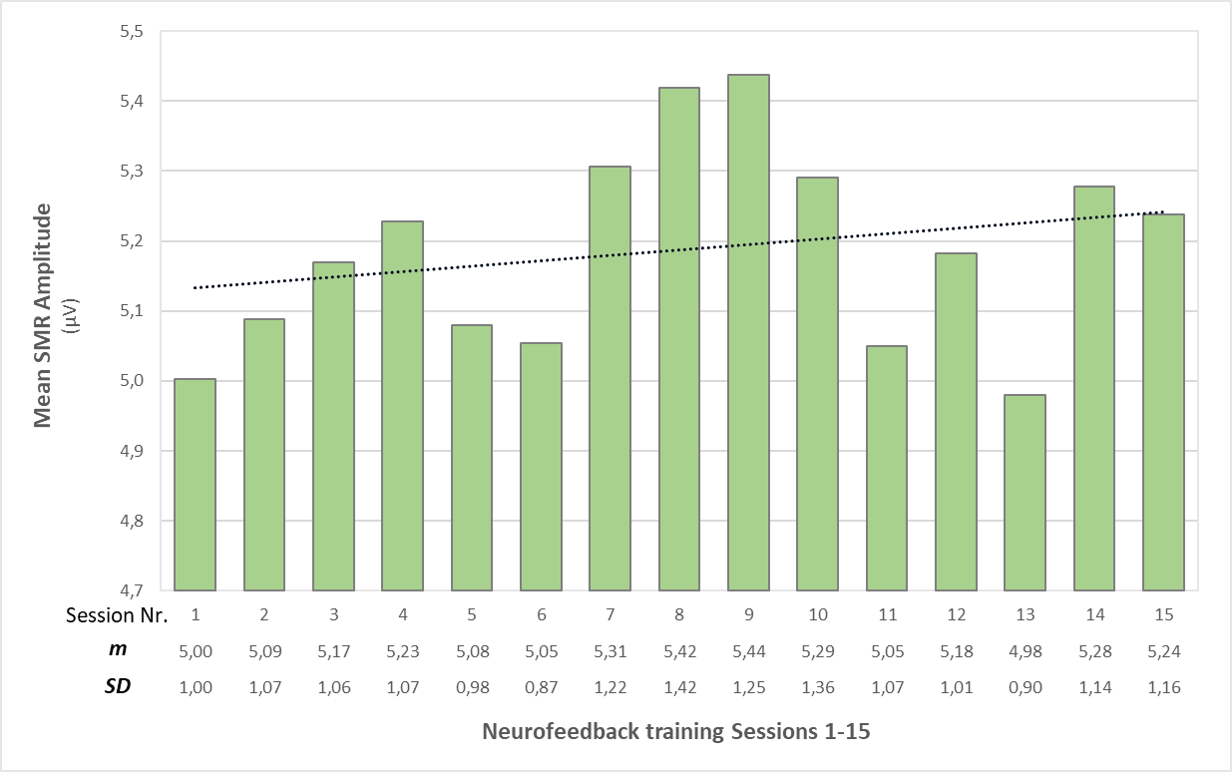

Supplement: nsae048_Supp [file nsae048_supp.zip › scan-23-223-File012.tif]

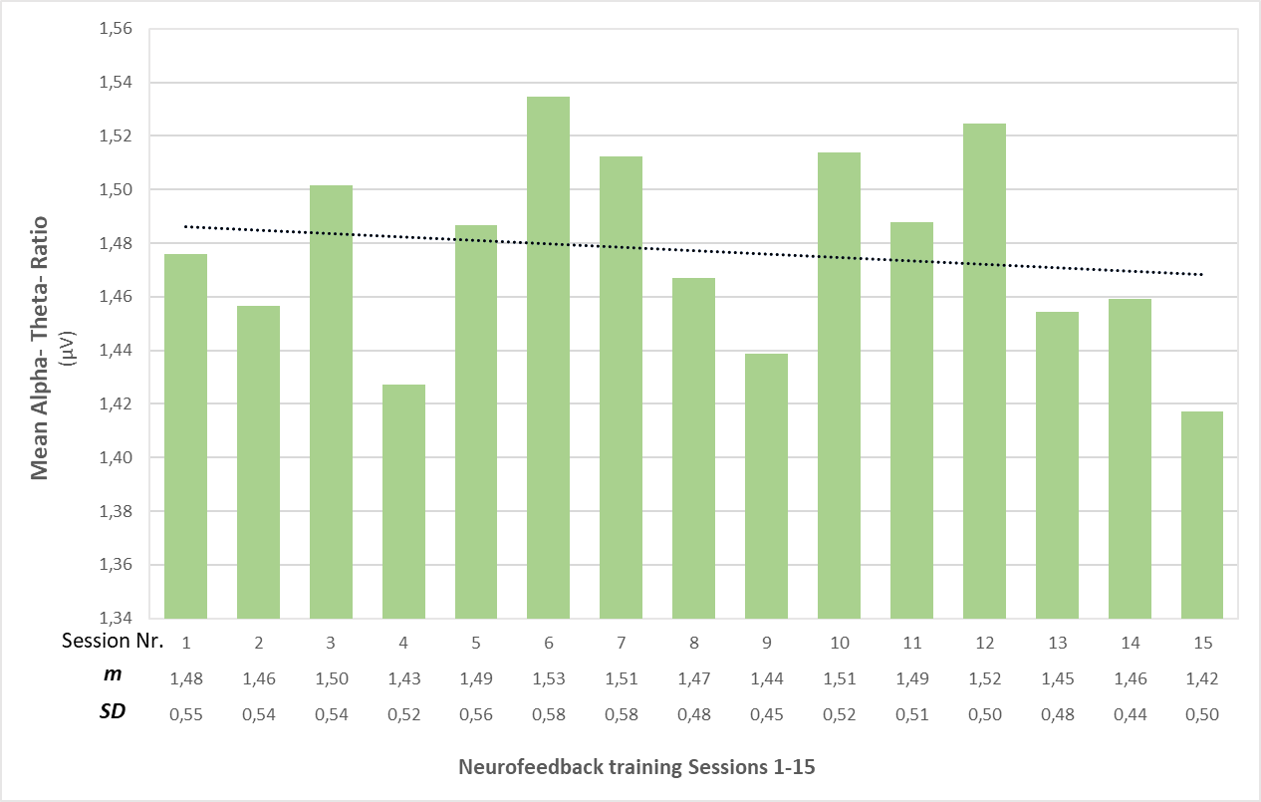

Supplement: nsae048_Supp [file nsae048_supp.zip › scan-23-223-File013.tif]
